# Fluorescence microscopy tensor imaging representations for large-scale dataset analysis

**DOI:** 10.1038/s41598-020-62233-2

**Published:** 2020-03-27

**Authors:** Claudio Vinegoni, Paolo Fumene Feruglio, Gabriel Courties, Stephen Schmidt, Maarten Hulsmans, Sungon Lee, Rui Wang, David Sosnovik, Matthias Nahrendorf, Ralph Weissleder

**Affiliations:** 1 Center for Systems Biology, Massachusetts General Hospital and Harvard Medical School, Boston, MA USA; 2 Department of Neuroscience, Biomedicine and Movement Sciences, University of Verona, Verona, Italy; 3 School of Electrical Engineering, Hanyang University, Ansan, Republic of Korea; 4 Department of Population Medicine, Harvard Pilgrim Health Care Institute and Harvard Medical School, Boston, MA USA; 5 Department of Biostatistics, Harvard T. H. Chan School of Public Health Center for Systems Biology, Massachusetts General Hospital and Harvard Medical School, Boston, MA USA; 6 Cardiovascular Research Center, Cardiology Division, Massachusetts General Hospital, Harvard Medical School, Boston, MA USA; 7 Martinos Center for Biomedical Imaging, Department of Radiology, Massachusetts General Hospital, Harvard Medical School, Boston, MA USA; 8 Cardiovascular Research Center, Massachusetts General Hospital and Harvard Medical School, Boston, MA USA; 9 Department of Systems Biology, Harvard Medical School, Boston, MA USA

**Keywords:** 3-D reconstruction, Fluorescence imaging, Optical imaging, Single-cell imaging

## Abstract

Understanding complex biological systems requires the system-wide characterization of cellular and molecular features. Recent advances in optical imaging technologies and chemical tissue clearing have facilitated the acquisition of whole-organ imaging datasets, but automated tools for their quantitative analysis and visualization are still lacking. We have here developed a visualization technique capable of providing whole-organ tensor imaging representations of local regional descriptors based on fluorescence data acquisition. This method enables rapid, multiscale, analysis and virtualization of large-volume, high-resolution complex biological data while generating 3D tractographic representations. Using the murine heart as a model, our method allowed us to analyze and interrogate the cardiac microvasculature and the tissue resident macrophage distribution and better infer and delineate the underlying structural network in unprecedented detail.

## Introduction

Comprehensively understanding tissues requires the integration of cellular information across multiple scales^1^. Recently developed optical clearing technologies^2–9^ as well as mapping approaches^10,11^ can now explore tissues at the single-cell level and provide an opportunity to better understand how tissue organization influences cellular function. These methods include tissue and organ clearing^8,9^ as well as mapping approaches^10,11^. Together, these methods may ultimately provide a global framework for comprehensively mapping the human body at a cellular resolution, thereby leading to deeper multidimensional descriptions of individual cells within their functional and 3D tissue contexts^1^.

As clearing technologies have advanced, so has the ability to profile larger tissue volumes from a few cell layers^12^ up to entire organs^1,13–16^. This development has created enormous computational challenges: manual segmentation, annotation and classification are no longer practical. Similarly, simple visual representation of these large, complex datasets at the organ level has been extraordinarily challenging as information spans several scales^17,18^.

Even though visualization is a recent research field which is a consequence of the modern scientific computing and computer graphics, it has been extensively increasing in importance and as pointed out by Walter *et al*.^19^ its use extends beyond what is the mere presentation of the imaging data. Visualization has become an invaluable tool greatly employed in research areas such as, for instance, medical, biological, and natural sciences. What is particularly difficult to achieve in computer visualization is to provide the user with the right representation of the salient information concealed in the data and to facilitate meaning extraction out of it. This point becomes even more problematic when data increase in dimensionality and size. New tools and resources to query and analyze such large-scale image data and to translate them into intuitive visual representation are therefore in high demand^19^ with the final goal to facilitate addressing broader biological questions^19^.

Tensor field visualization in particular is an important visualization technique in imaging as it allows the user to depict the order in data starting from a local description of a sensed quantity which is better represented by a tensor.

Tensors are sophisticated mathematical entities whose implementation has broadly increased during the last decades, in particular in the imaging and visualization field. As an extension of scalars and vectors, they are more suitable to describe and characterize physical quantities which are difficult to represent otherwise. So far they have been largely used to accurately describe classical physics phenomena spreading from electromagnetism (e.g. polarization tensor, EM tensor) to mechanics (e.g. tensor of inertia, stress tensor) as well as others. Tensors find also applications in fluid dynamics, the theory of relativity and amongst several computer science and engineering fields. Also machine learning, computer graphics and vision, just to name a few, take advantage of the use of such a mathematical tool.

Specifically in the field of medical imaging, tensors are used to describe the molecular diffusion within a tissue, mainly through DTI-MRI scans. As water diffusion displays an anisotropic behavior, which is adequately described by a 3 by 3 second rank tensor, it is used to distinguish nerve bundles within the brain and other organs such as the heart where it is possible to reconstruct the myocytes myocardial paths. Since in DTI it is possible to directly associate at each voxel a tensor entity, several display and visualization methods have been developed. Many of these methods rely on the representation of a tensor as an ellipsoid whose axes are aligned along the tensor eigenvectors and with length proportional to the corresponding eigenvalue.

Also, due to the capability of DTI to capture neural fibers directionality, a class of algorithms has been introduced and utilized to perform fiber tractography. Through tractography, cardiac myofiber architecture has been detected^20^ and it has been used to gain a new understanding of the underlying mechanics or to give rise to mathematical models.

Due to the intrinsic capabilities of tensors to characterize and identify various structural information and inspired by work in the DTI-MRI field, we have here extended the applications of this tool to fluorescence microscopy to gain insights into very large and dense datasets that would be otherwise difficult to both decode and display.

Here we introduce a fluorescence microscopy tensor imaging method for analyzing and virtualizing fluorescence microscopy organ datasets. This method consists in extracting from binarized imaging datasets, a set of morphological descriptors based on specific biological questions. These are then used to build a local voxel-wise variance-covariance matrix to obtain a volumetric tensor-value representation of the imaging dataset. Finally, local image correlation is characterized and salient local geometrical information is extracted and visualized. The process is iterated among any sub-volumes giving rise to a tensor field to express relevant image information. As descriptors and voxel size are in general user-defined parameters, our method is characterized by a great flexibility, which reflects on the possibility of creating 3D representations at different resolution scales and in a multifactorial fashion.

The method is a powerful tool for complete virtualization and synthesis of imaged samples’ geometry and topology as well as fiber tract maps. The use of the term fiber is here intended to represent “bundles of organizational tracts”. Tractography maps of the microvasculature and macrophages’ organization can be obtained by visualizing “streamlines of organizational tracts”. The method’s implementation on currently available large-scale dataset can allow for unprecedented insights at many levels of biological scale. It is also independent from the chosen segmentation approach, it could be seamlessly applied to freely available digitized atlases, and it could synergize with several existing computational approaches and acquisition modalities such as micro-XCT, synchrotron radiation phase contrast imaging, and histopathology. Here we apply our method to mapping of the murine heart and we demonstrate how organizational tracts orientations and transmural angle maps can be resolved in greater details.

The method could be also applied for visualization of large *in vivo* data collections such as dorsal window chambers or in developing zebrafish.

## Results

### Data acquisition and segmentation

To demonstrate our imaging method on real data we needed first to acquire a whole organ imaging dataset and then perform accurate binarization for extracting the sets of morphological descriptors from which the volumetric tensor-value representation can be generated. Supplementary Fig. S1 summarizes the general acquisition and processing pipeline. We here focused on the cardiac microvasculature as it is highly relevant to organismal health, well understood and can be easily validated, but other organs could be also considered.

**Figure 1:**
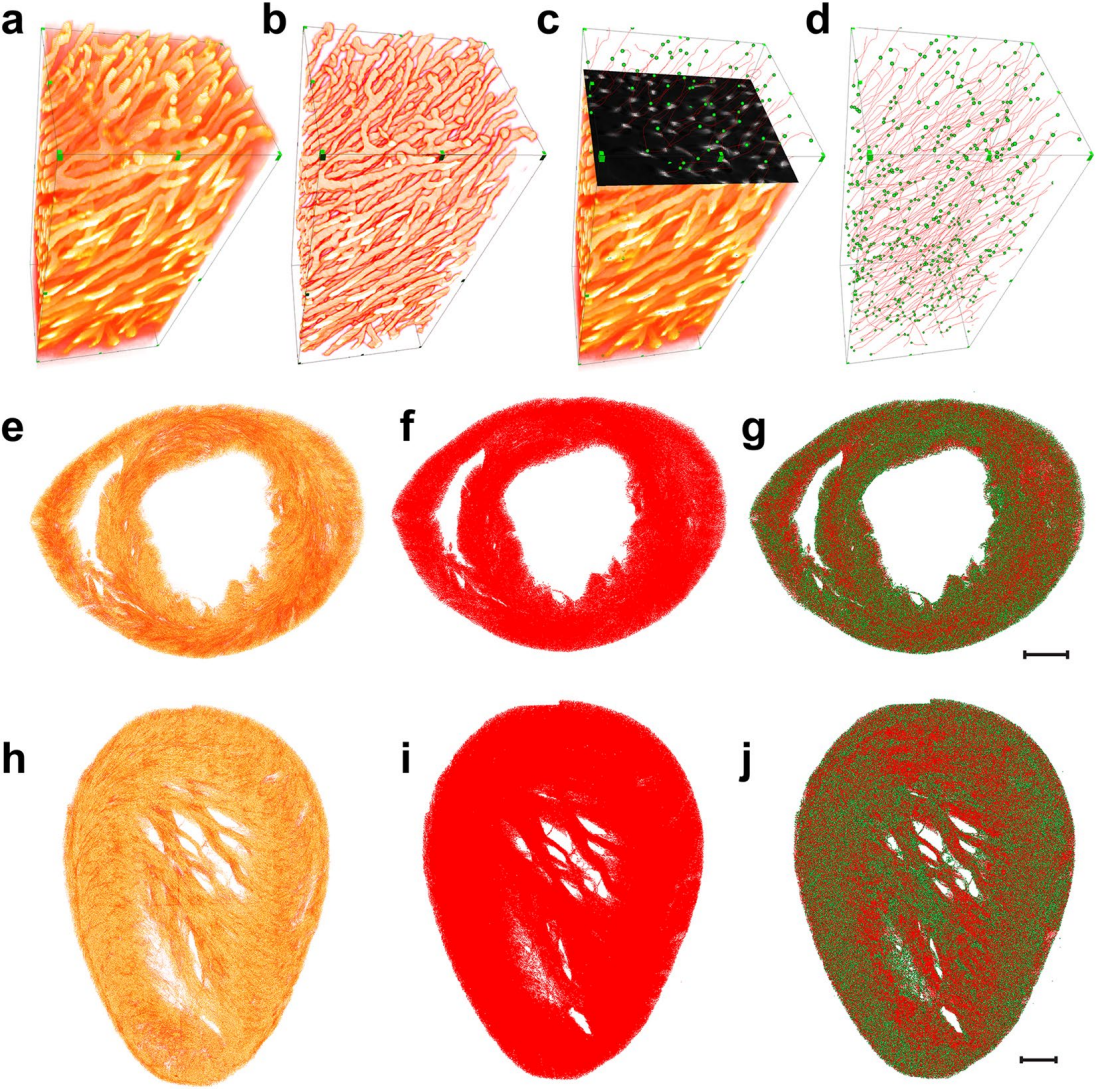
Heart microvasculature acquisition. 3D rendering of a representative volume of lectin-TRITC stained heart microvasculature. Comparison between the raw data (**a**) and the ML-based segmentation results (**b**). (**c**,**d**) Automated feature extraction of the vascular skeleton (red lines) with endpoints and bifurcations represented as nodes (green circles). Bounding box, 105 × 120 × 345 μm. (**e–g**) 3D rendering of a representative short-axis basal slice with fluorescence microvasculature signal (**e**), microvascular skeleton (**f**) and nodes (**g**). (**h–j**) 3D rendering of a representative sagittal slice with corresponding microvascular skeleton and node representations. Scale bars, 500 μm.

After sample preparation and optical clearing (see Supplement 1), images were acquired by fluorescence confocal microscopy at sub-cellular resolution. Images were then computationally pre-processed to maximize signal-to-noise ratio (SNR) and contrast ratio (Supplementary Fig. S2, Fig. 1a) (see Supplement 1). Because the dataset is volumetric in nature, we used 3D segmentation algorithms to properly segment the microvasculature (Fig. 1b). Due to the large size and complexity of typical organ datasets, traditional methodologies based on manual tracing have become impractical. We therefore implemented custom-designed, supervised neural networks (Supplementary Fig. S3), with parameters determined through manually annotated datasets (see Supplement 1). The goodness of the automatic segmentation procedure compared to a manually obtained one (also determined through the use of Dice coefficient as given in Supplement 1) is visually shown for several volumes (Supplementary Fig. S4) in Supplementary Figs. S5 and S6 and in Supplementary Movies 1–6.

Since structural and topological analysis (e.g. complete graph, nodes, links, bifurcations, vessels length, etc.) can be readily obtained from the centerline information, extracting skeletonization after binary segmentation (Fig. 1c,d and Supplementary Movie 7) generated a symbolic description of the dataset. This processing extracts region-based shape features through so-called thinning algorithms. Whole heart slices were then processed, and both microvasculature and graph representations were obtained (Fig. 1e–j).

What clearly emerges from the obtained datasets is the high complexity of the structural properties and the topology of the vascular network (Fig. 1g,j, Supplementary Movie 8) which makes it hard to infer and delineate what is the overall underlying vascular structure.

### Mathematical framework

**Figure 2:**
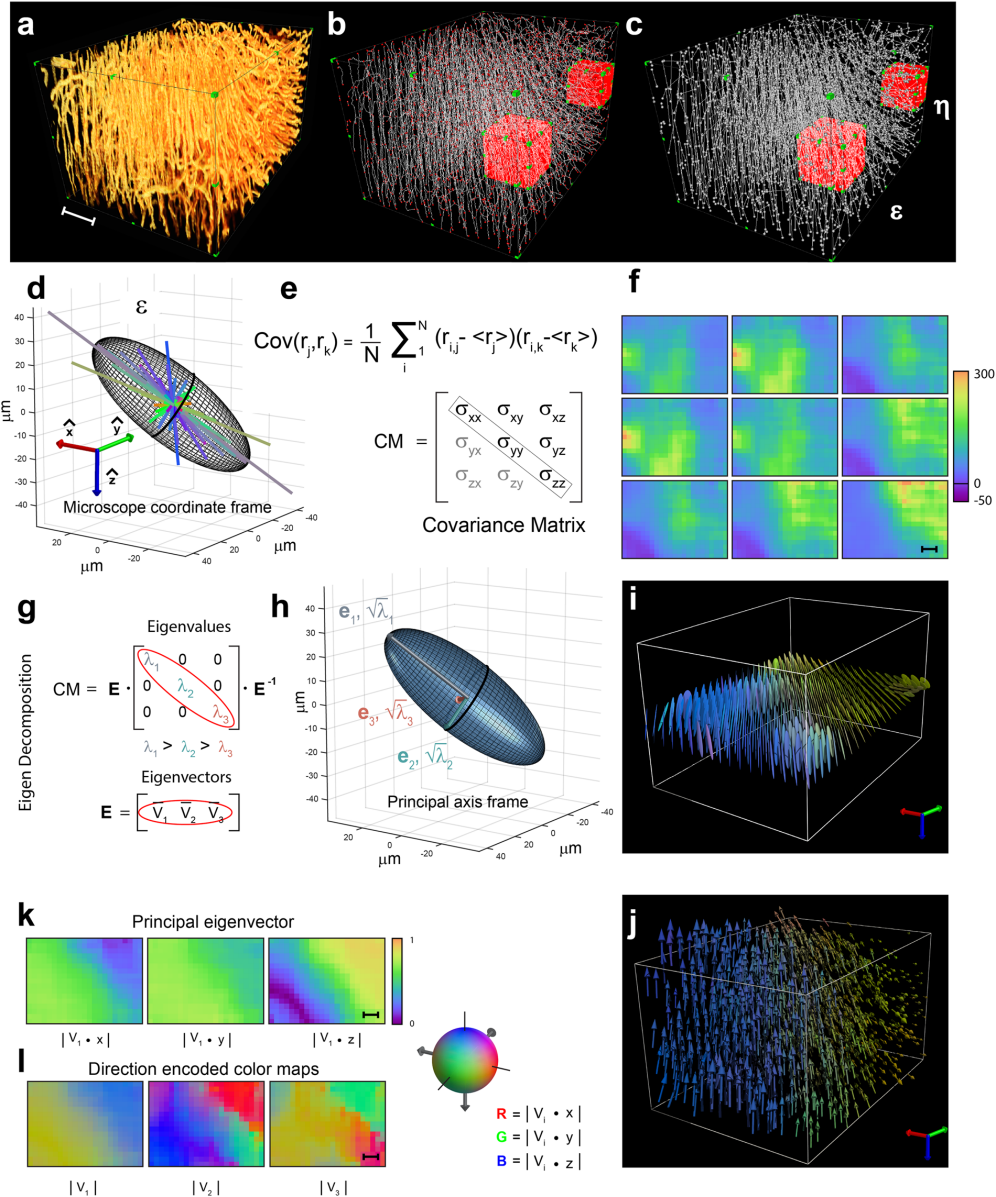
Covariance matrix and tensor imaging representation. 3D renderings of a representative volume of lectin-TRITC stained heart. (**a**) Image processed fluorescence data, (**b**) corresponding skeleton (white) and graph nodes (red), (**c**) nodes (white circles) and their connecting segments (white lines). Bounding box, 250 × 205 × 340 μm. (**d**) 3D representation of the covariance ellipsoid. All feature descriptors (segments, for the microvasculature case) present within the red sampling volume ε (corresponding to a voxel in the final 3D image) are plotted and rendered with a directionally encoded color, defined in the microscopy acquisition frame. (**e**) By calculating the covariance Cov of the vascular tracts we can obtain the entries of the variance-covariance matrix CM (3 × 3), which is a tensor of rank 2. Here the matrix’s diagonal entries are the variances, while the others are the covariances. (**f**) Image representation of the tensor field components of the mid axial plane of the representative volume of lectin-TRITC stained heart shown in (a), using a pseudo-color map. Off diagonal components rescaled for better contrast. (**g**) Rotating the reference frame makes it possible to diagonalize the CM tensor with the positive eigenvalues λ_1_, λ_2_, λ_3_ related to the spread (variance) in the eigenvectors directions v_1_, v_2_, v_3_. (**h**) In the new principal axis frame, the principal eigenvector v_1_ gives the main vascular directionality within every voxel. The square root of the eigenvalues is proportional to the ellipsoid radii. The eigenvectors to their orientations. (**i**) The 3D ellipsoid glyph-based visualization of the mid axial plane of the representative volume of lectin-TRITC stained heart shown in (a), describes the tensor information at any point along an axial plane of the microvasculature. Ellipsoid colors are rendered using a directional color-encoded map (X, red; Y, green; Z, blue). (**j**) 3D vector-field representation of the entire vascular volume in (**a**). (**k**) Voxel maps of the principal eigenvector component along the lab reference frame, using a pseudo-color map, for the mid axial plane of the representative volume of lectin-TRITC stained heart shown in (a). (**l**) Directional color-encoded maps of the CM eigenvectors for the same mid axial plane shown in (k). RGB components are defined as the absolute values of the eigenvectors’ XYZ components (see inset). Scale bars in (**f**,**k**,**l**), 50 μm.

The mathematical framework at the core of our method is here illustrated. Within a sampling volume (voxel) of an imaging dataset, a set of morphological feature descriptors can be extracted starting from the segmented raw data (Fig. 2a–c, Supplementary Movies 9 and 10). These descriptors are user-defined depending on the specific biological problem and are the result of different image processing techniques. For imaging capillary’ structures, one possible feature descriptor of interest could be the vascular branches’ specific spatial orientation and length (Fig. 2c). Conversely, for individual cells, regional density heterogeneity, cellular orientational axis or morphological phenotypes may reflect steady state function in healthy tissue or pathological changes in diseases such as heart failure.

An effective method to quantify the spatial patterns and characterize the overall directional distributions of the descriptors ***r***_*i*_ (in general vectors) within the defined voxels (red boxes in Fig. 2c) relies on statistical representations such as the standard deviation ellipsoid (Fig. 2d, Supplementary Fig. S7), which is an isosurface of directional probability. In Fig. 2d all feature descriptors ***r***_*i*_ present within the red sampling volume ε (corresponding to a single voxel in the final 3D image) and corresponding to the vascular branches present in it, are plotted and rendered with a directionally encoded color, as defined in the microscopy acquisition frame.

We next calculate the variations and covariations of the descriptors’ coordinates from their mean in each voxel (Fig. 2e), thus capturing the local distribution. If this spread is homogeneous, the data can be conceptualized as a sphere since no particular dimension prevails over another (isotropic). Conversely, when the spread is heterogeneous, the data correlate and their distribution is better expressed as an ellipsoid with a particular spatial orientation and axes of different sizes (anisotropic). The approach is similar to diffusion tensor imaging (DTI) MRI^21,22^, though DTI-MRI attains diffusion ellipsoid shape and orientation from separate, distinct diffusion measurements made along multiple independent directions, while our method extracts information after binarizing the data. This allows us to extract several feature sets that are independent of the acquisition modality and to build virtual mathematical models that provide multiple ways to synthesize the local cellular architecture.

Mathematically it is convenient to express the standard deviation ellipsoid as a second order tensor (Fig. 2e), because spheres and ellipsoids can be easily represented by such a matrix (CM), which in turn can be directly derived from the voxel data. In our case, the tensor is a 3 × 3 matrix and captures the basic features of the dataset’s local geometrical structure. In particular, the tensor encapsulates information about the scattered data’s orientation and spread within the voxel of interest. Its axes are oriented along the direction of maximum variability and the axes’ sizes correlate with the spread’s magnitude.

The ability to assign a tensor representation for all feature descriptors present within each voxel is important due to the tensor’s further statistical interpretation, which permits us to draw a bridge with the geometrical ellipsoid representation. In fact, the 3 × 3 matrix can be contextualized in multivariate statistics, particularly as the variance-covariance matrix^23^ in three-dimensional Gaussian distributions. The individual matrix entries are the result of computing all possible covariances among all feature descriptors’ components ***r***_*i,j*_ (*i* = 1*. N*, *j* = *1–3*) with respect to the microscope reference system. Here *N* represents the number of elements per voxel, while *j* runs over the 3D space dimension (*X*, *Y* and *Z* components). The tensor diagonal components are represented by the terms *cov(X,X)*, *cov(Y,Y)* and *cov(Z,Z)* which are particular cases as they coincide with the variances of *X*, *Y* and *Z* respectively (Fig. 2e). Under our assumptions, the matrix is symmetric positive definite, and therefore only six entries are necessary when dealing with three dimensions (Fig. 2e,f).

When the ellipsoid aligns with the main frame axes (microscope reference frame), only the principal diagonal entries of the 3 × 3 correlation matrix are non-zero (diagonal tensor). Specifically, these eigenvalues are the values obtained by computing variances of the *x*, *y* and *z* data components, respectively, and are proportional to the squares of the lengths of the ellipsoid axes^24^. Non-diagonal entries, by contrast, produce a rotated ellipsoid, in which case the covariance matrix univocally defines the ellipsoid spatial orientation and axis lengths.

The spectral theorem^25^ guarantees that it is possible to diagonalize the non-singular cross correlation matrix (Fig. 2g) and determine an orthogonal basis (eigenvectors) that defines the principal coordinate directions, with dimensions and eigenvalues equal to the matrix size. Importantly, this allows one to characterize the structural properties independently from the microscope acquisition frame and to work within each voxel in a local coordinate system solely determined by the specimen anatomy. In this principal frame, the ellipsoid’s axes are aligned along the eigenvectors of the variance-covariance matrix, and the ellipsoid axes’ lengths are regulated by the eigenvalues’ magnitudes^24^ (Fig. 2h).

The above procedure and interpretations can be unified into a single framework, i.e. the principal component analysis (PCA)^26–32^, which is commonly used to reduce large datasets’ dimensionality^33,34^. By exploiting the variance-covariance matrix computed from the data, eigenvectors and eigenvalues are calculated and a change in the data coordinate system is introduced such that the projected feature points’ greatest variation occurs across the new axis.

The eigenvector with the largest eigenvalue (*v*_*1*_) corresponds to the dimension with the strongest correlation, i.e. principal component (PC), while the corresponding eigenvalue represents its variability. The second eigenvector (*v*_2_) represents the maximum variability along a direction orthogonally oriented with respect to the first one. The third (*v*_3_) is the maximum variability orthogonally oriented with respect to the previous two.

Because each voxel corresponds to a specific tensor that contains six degrees of freedoms (in addition to the three spatial), volumetric datasets can be associated with tensor fields (TF), or collections of region descriptors, which reflect the order on a unique local orthogonal coordinate system and produce tensor-valued images (see Supplementary Information). For an intuitive representation of the tensor data for an axial slice of the tensor field, see Fig. 2f. One challenge is how to best interpret and visualize such highly dimensional data^35,36^. Possible approaches include tensor glyphs (Fig. 2i, Supplementary Movie 11), i.e. 3D parameterized graphical objects representing the different characteristics of each tensor point with shape, size, position and color, respectively^37^. In this representation, the eigenvectors define the local voxel glyph spatial orientation, while the eigenvalues define the glyph lengths. Other interpretations based on vector fields (VF)^38^ (Fig. 2j), 2D scalar maps (Figs. 2k,l, and S5a–f) and tractographic representations are also possible^39^ ways to visualize data.

**Figure 3:**
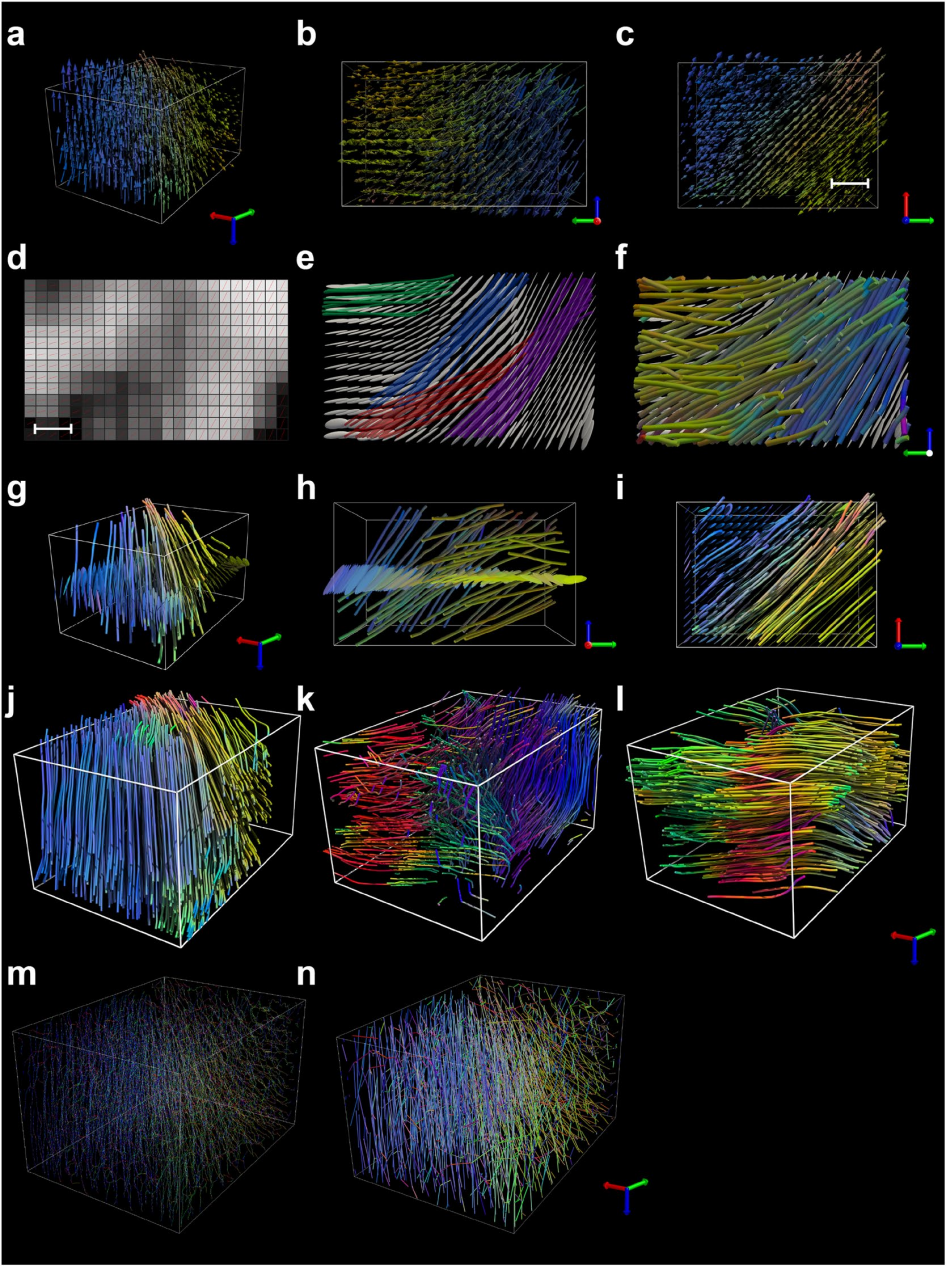
Content visualization and vascular tractography. All representations and visualizations refer to the representative volume of lectin-TRITC stained heart shown in Fig. 2(a). The 3D perspective (**a**), axial (**b**) and sagittal (**c**) vector field representations of the primary eigenvector clearly show the spatial preferential orientation of the microvascular network present in the tissue volume of Fig. 2a. Vectors are color coded to represent orientation with respect to the *XYZ* lab frame. (**d**) Map of the principal eigenvector along a sagittal plane (Supplementary Fig. S7g–h) of the volume in (**a**). The intensity corresponds to the principal eigenvector’s length, with hedgehogs representing its direction in space at each point in the field. (**e**) Ellipsoid glyph representation of the tensor field for the same sagittal plane. Different streamline groups represent different trajectories along the principal eigenvector direction. (**f**) Color-coded fiber tracts represent seeded streamlines along the principal eigenvector and illustrate the main distribution and direction of the microvasculature. 3D perspective (**g**), axial (**h**) and sagittal (**i**) vascular tractograms with an associated axial ellipsoid glyph representation. (**j–l**) Tomographic vascular tractograms obtained along the principal (**j**), secondary (**k**) and tertiary (**l**) eigenvector, respectively. 3D rendering of the vascular skeleton (**m**) and the segments connecting the graph nodes. Color coded, *RGB*. Bounding box, 250 × 205 × 340 μm. Scale bars, 50 μm.

The tensor field^40^ information can be reduced^41^, assuming the principal eigenvectors encode the locally prevailing vascular orientation in each voxel (Fig. 3a–c).

The accompanying eigenvector field representing the fiber orientation maps (FOM) (Fig. 2j) can be converted to a color space representation^42^, to build 2D maps for more immediate interpretation of the underlying 3D vascular or cellular structure (Figs. 2k,l and 3d, Supplementary Fig. S8). A directionally encoded color scheme (DEC)^43,44^ defined in the microscope acquisition frame can visually facilitate tensor interpretation. Other eigenvalue-derived tensor metrics include the fractional anisotropy (FA) (Supplementary Fig. S8f), which encodes directionality component strength, or the Westin’s coefficients^42^ (Supplementary Fig. S8b). Using the eigenvectors’ orientation and a tensor-based deterministic method based on the 2^nd^ order Runge-Kutta method^45^, 3D trajectories can be also reconstructed (Fig. 3e–l) (see Supplementary Information). Directional tracking through the eigenvector fields is obtained by propagating the tracks in a voxel-by-voxel manner from selected seeds (Fig. 3e) while following the eigenvectors’ local orientation (Fig. 3f). The associated fiber bundles (Fig. 3g–l, Supplementary Movie 11) give insights into the underlying anatomical structures and their regional variations and trends (Fig. 3m,n). Indeed, by varying the sampling volume over which the PCA analysis is performed, it is possible to provide a mathematical description at virtually any scale of magnification.

### Fluorescence microscopy tensor imaging representations of the cardiac microvascularity

**Figure 4:**
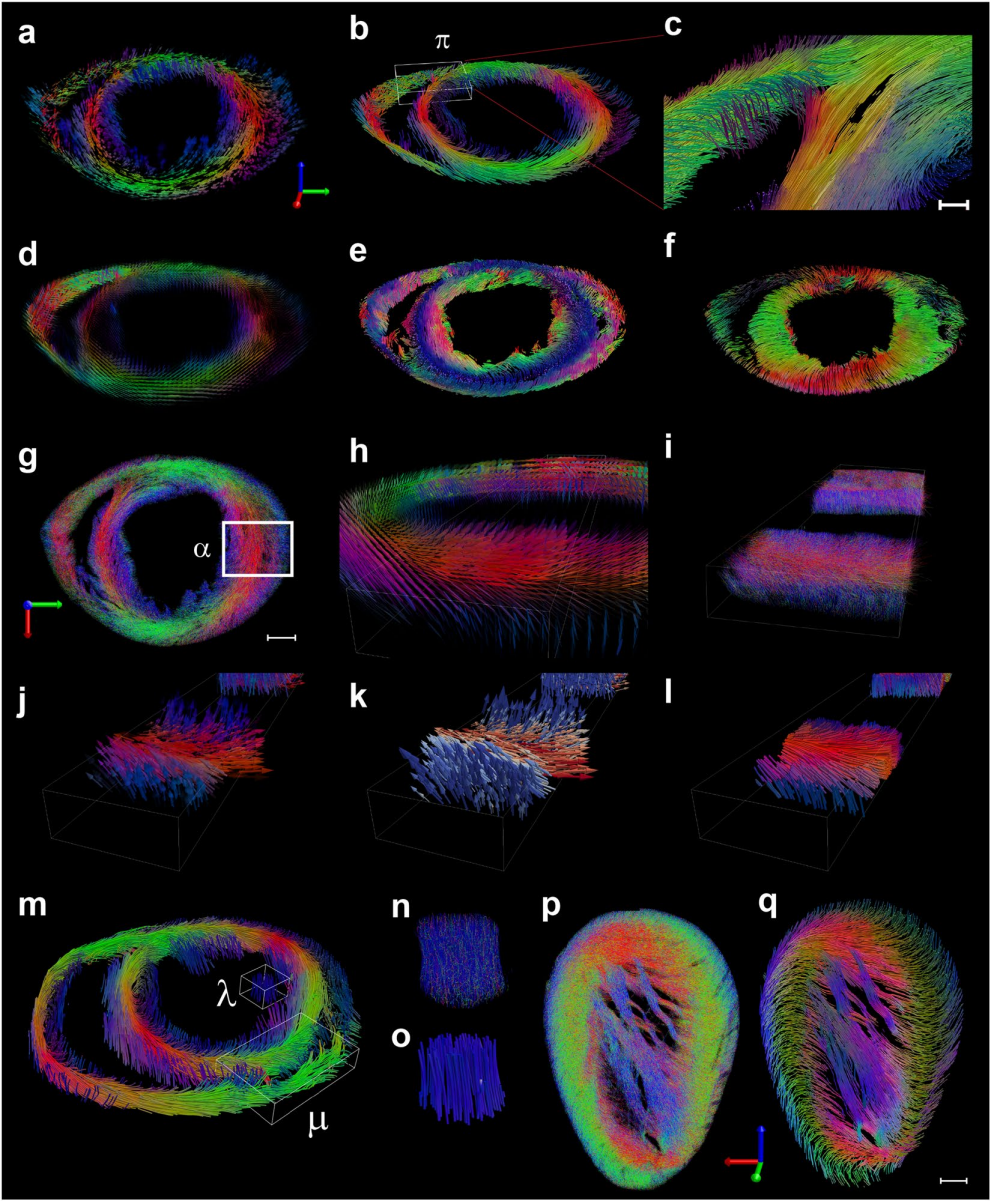
Tomographic vascular tractograms. (a-l) Representations and visualizations of the microvasculature for a lectin-TRITC stained apical short-axis slice (as shown in Fig. 1e). (**a**) Tomographic vector field representation of the vascular directional primary eigenvector in a lectin-TRITC stained apical short-axis slice (as shown in Fig. 1e and plane 1 of Fig. S13). Tomographic vascular tractogram determined along the primary eigenvector (**b**) and magnified view of the posterior left/right ventricle border area (**c**). Scale bar, 100 μm. (**d**) 3D ellipsoid glyph representation of the tensor field for the representative slice in (**a**). (**e**,**f**) Tomographic vascular tractograms determined along the secondary (**e**) and tertiary (**f**) eigenvectors. (**g**) Skeleton axial view representation of the vascular network. (**h–l**) 3D magnified views of the area α To (**g**). Glyph tensor field representation (**h**) and vascular skeleton (**i**) with corresponding vector field representations (**j**,**k**). In (**j**) colors encode directional information, while in (**k**) a cold-hot pseudo-color map encodes the principal eigenvector projection’s magnitude along the vertical axis. (**l**) 3D rendering of the fiber tracts corresponding to the magnified area. (**m**) Tomographic directional vascular tractogram in a lectin-TRITC stained basal short-axis slice (plane 3 of Fig. S13). (**n**,**o**) Magnified skeleton representation of the microvasculature in *λ* and corresponding tractogram. (**p**,**q**) Skeleton representation of the microvasculature and corresponding tractogram for a representative lectin-TRITC stained sagittal slice (as shown in Fig. S1h). Scale bar, 500 μm.

We then used our method to interrogate the cardiac microvasculature and obtain structural tractographic representations in unprecedented detail. We first transformed the segmented vascular imaging datasets (Fig. 1e–j) into completely virtualized tensor field representations. To quantify local variations within these representations and characterize the different vascular myocardial features, the vascular tensor fields can be simplified to vector field representations defined along the principal eigenvectors that here represent the vascular directionality. Figure 4a (Supplementary Movie 12) shows a tomographic VF representation of the vascular directional primary eigenvector in an apical short-axis slice (mid and basal short-axis representative slices are provided in Supplementary Fig. S9). To represent the major eigenvector’s spatial orientation we use a direct *RGB-to-XYZ* mapping color scheme (Fig. 2). The red color indicates right-left orientation, green the anterior-posterior orientation and blue the superior-inferior orientation. Together, the VF and corresponding tractogram (Fig. 4b, Supplementary Movie 12) emphasize the left-handed helical orientation of the microvasculature in the left ventricular wall and the myocardial fibers’ overlapping course in the transition area from epi- to endocardium (Supplementary Fig. S9). A detailed magnified view (Fig. 4c) of the septal boundary at the intersection of the left ventricle and lateral boundaries shows intricate vascular structure branching near the interventricular sulcus. Two-dimensional directional encoded colored (DEC) maps of eigenvectors, tensor field representation and scalars detail the local 3D vascular organization (Supplementary Fig. S10). A tensor glyph representation (Fig. 4d) provides visual insights into the complex tensor field at each voxel to better emphasize local order and directionality, particularly along directions different from the principal ones to interrogate myocardial vascular sheet structures^46,47^.

The high level of structural hierarchy can be further analyzed by comparing the overall organization present within the vascular tractograms determined along the secondary (sheet-parallel) and tertiary (sheet-normal) eigenvectors, respectively (Fig. 4e,f), in conjunction with the original vascular skeleton view representations (Fig. 4g). Overall, the VF and tractograms emphasize how the vascular network presents a gradual counterclockwise rotation similar to the classical myocardial fiber orientation from the epicardium to the endocardium. The directional color-encoded maps of the principal eigenvectors (Supplementary Fig. S10) together with their corresponding vascular tractograms (Fig. 4b,e,f), are in good agreement with results obtained by diffusion tensor MRI^20^ and structure tensor synchrotron radiation imaging^48^, while offering a much higher degree of details.

In the epicardium and endocardium, the primary eigenvectors are mostly oriented in the longitudinal apex-base direction. In the mid-wall, the fibers are oriented predominantly in the circumferential direction. This is better emphasized in the magnified views of the lateral exterior left ventricle (Fig. 4h–l). The effect can be quantified along the transmural depth, from epi- to endocardium, using a new set of coordinates defined by the transverse and helix angle (HA) relative to the local wall (Supplementary Fig. S11). In this frame, vascular fibers with positive helix angle values run toward the antero-apex direction, while those with negative values run toward the postero-apex one. Plots of the helix angle as a function of the transmural distance^49^ indicate that vascular tracts in the subepicardium and subendocardium have positive and negative helix angle values, respectively, giving rise to a local helical architecture presenting a transmural angle of 150 degrees (Supplementary Fig. S11f). Tracts in the midcardium, where the helix angle approaches null values, are strongly circumferential (Supplementary Fig. S9d–g).

Tomographic vascular tractograms of a representative basal short axis slice (Fig. 4m, Supplementary Movie 13) reveal the mainly vertical pathways of the vascular fiber tracts within the papillary muscles (blue color-coded, along the inferior-superior orientation) (Supplementary Fig. S12). Representative vascular skeletons and tractograms of sagittal slices are shown in Fig. 4p,q and Supplementary Movie 14. Vascular tracks can be seen spiraling down the apex, following the general layout of cardiomyocytes that are spatially organized to optimize the rhythmic cardiac contractions that lead to blood ejection into the aorta. This process is highly energy dependent, requiring ample oxygenated blood and nutrient supply through capillaries that are in close proximity to contracting cells, a requirement fulfilled by the similar orientation of muscle fibers and the vascular bed.

DTI cannot directly image the heterogeneity of the fiber tracts orientations within a single voxel (typically 100 microns isotropic). To validate our results we have therefore used diffusion spectrum magnetic resonance imaging (DSI)^20^ (see Supplementary Information), due to its ability to image intravoxel fiber tract intersections at resolutions more realistically comparable to the ones obtained with our method. A direct comparison between helix angle representations obtained with our method (Supplementary Fig. S11d) and with DSI (Supplementary Fig. S11g), shows that the two modalities are in good agreement with each other. Clearly our method provides informative maps at much higher resolution and allows to resolve in greater details the tracts orientations and to accurately follow the gradual change of the helix angle from the epi- to the endocardium (Supplementary Fig. S11f). Also the measured value (150 degrees) of the transmural angle (Supplementary Fig. S11f) is in good agreement with DSI obtained values^20^.

The location within the heart where the axial slices have been taken is shown in Supplementary Fig. S13. A flowchart describing the elaboration and visualization process is given in Supplementary Fig. S14, with a description of the visualization tools used.

### Cellular orientation of cardiac tissue resident macrophages

**Figure 5:**
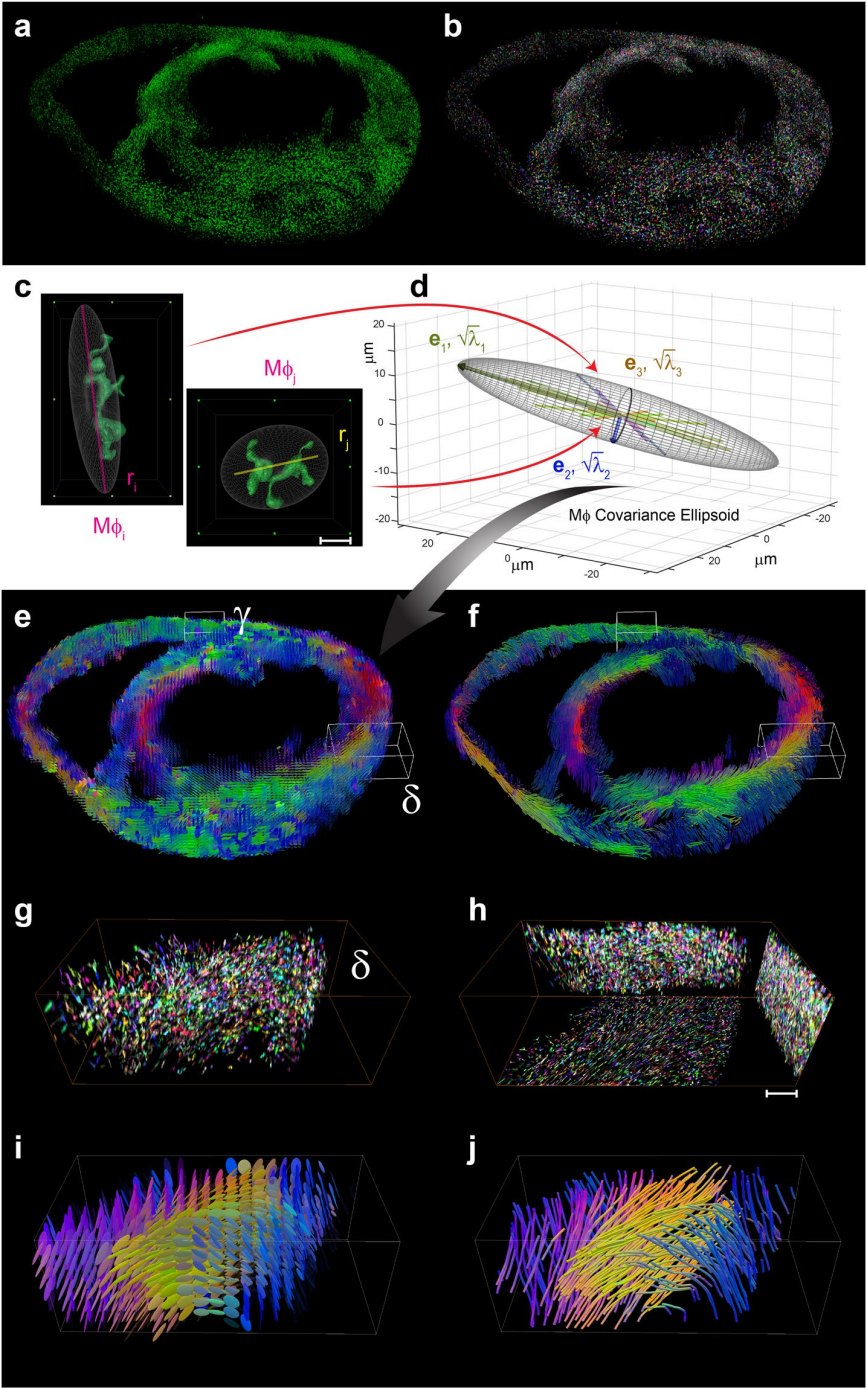
Tomographic cellular tractograms. (**a**,**b**) 3D renderings of the anti-GFP stained GFP-expressing macrophages distribution present in a representative basal short-axis slice. (**a**) Raw fluorescence signal. (**b**) Cells are segmented and randomly color coded for better visual discrimination. (**c**) Magnified views of two different macrophages and their spatial distribution, with the fitting ellipsoids and their respective principal axes (feature descriptors). Scale bar, 10 μm. (**d**) 3D representation of the cellular covariance ellipsoid Mφ, which represents the isosurface of the macrophages’ directional probability on a single voxel. (e-j) Representations and visualization of the anti-GFP stained GFP-expressing macrophages distribution present in the basal short-axis slice shown in (a,b). (**e**) The 3D ellipsoid glyph-based visualization describes the macrophage tensor field information. (**f**) Corresponding directional cellular tractogram. (**g**,**h**) Magnified 3D rendering of the macrophage population in δ (**G**) and maximum intensity projections along orthogonal planes (**h**). Scale bar, 100 μm. 3D rendering of the ellipsoid glyph-based tensor (**i**) and respective cellular directional tractogram (**j**). Colors are rendered using a directional color-encoded map.

A second application of our visualization technique is its use in cellular mapping over whole organs. Here we chose to investigate tissue resident macrophages in the myocardium as these cells have important homeostatic, house-keeping and other newly appreciated functions^50,51^. We imaged the perfused and cleared hearts of Cx3cr1GFP/+ mice, a reporter strain in which green fluorescent protein (GFP) can be used to identify cardiac macrophages in the normal heart^50^. These cells, which have a central body and long protrusions that mingle in between other stromal cells and cardiomyocytes, have been shown to have important sensing functions^52^ and even play a role in electrical conduction^50^. Consecutive thick murine heart slices were stained with anti-GFP to amplify the fluorescent signal. We also experimented with GFP-preserving fixation methods^9^, although anti-GFP staining resulted in higher SNR. Volumetric datasets were then assembled and processed (Fig. 5a,b, and Supplementary Movie 15). For each macrophage we estimated the best-fitting ellipsoid and determined the ellipsoids’ principal axes, which are assumed to represent the individual cellular orientational axes (Fig. 5c). We then determined the statistical distribution of the descriptor vectors within a sampling volume by PCA (Fig. 5d). Finally, we propagated the computation of the macrophage covariance matrix over all the sampling volumes to infer the overall organ cellular spatial distribution. We found that cardiac macrophages do not randomly orient themselves but rather preferentially align with each other in distinct directions (Fig. 5e–f, Supplementary Movie 16). This orientation is similar to that observed for the microvasculature, a similarity emphasized in the magnified 3D renderings (Fig. 5g–j).

## Discussion

Our goal was to develop an imaging and computational method to automatically extract spatial, cellular and molecular information from large-size fluorescence microscopy imaging data sets. By using the feature vectors’ variance-covariance matrix as a local region descriptor^53^, we virtualized and obtained tensor field data representation. We have demonstrated how vascular information can be visualized regionally and throughout the murine heart. Since visualizing large datasets is critical to improving analysis, we also developed and applied different tensor metrics derived from both eigenvectors and eigenvalues. Our segmentation method of choice relied on a machine learning custom-based solution, but other methods are possible, and data acquired and segmented by other groups with alternative methods as well as freely accessible already digitized atlases, can be seamlessly integrated into our mathematical framework pipeline to generate tensorial multiscale maps.

Acquiring and navigating large datasets has been attempted in the past. OCT^54,55^, PS-OCT^56^, histopathology^57^ and other techniques have been used to map fiber orientation, but these approaches mainly used size-limited acquisitions and/or direct elaboration schemes. Examples of direct methods for fibers orientation mapping include those based on the Fourier transform^58^ and wedge filters^59^ as well as others based on image filters such as the gradient or the second order derivative information^60–63^. Generally, these methods are fast but require prior knowledge of the imaged sample features to identify the most relevant ones and to properly perform orientation estimation. Furthermore, these and other filtering-based approaches are intrinsically correlated to the analysis, which de facto reduces their flexibility, leading to high reprocessing times if other features or different sampling rates are required.

Alternatively, segmentation-based methods preserve the entire image information, thereby facilitating complete binarization of a sample volume. By dissociating the processing and analysis steps, this complete binarization permits more freedom regarding feature analysis and speeds up the analytical process, e.g. when multiple descriptors are considered at the same time. Moreover, this approach provides a correct simplification and data down-sampling without altering any geometrical or signal intensity characteristic of the original images.

While we here chose to apply our method on the murine cardiac microvasculature, our imaging method could be applied to other cellular structures within the heart for instance to determine the distribution and orientation of the macrophage population, or to other organs. In the brain for example it could be used in order to produce detailed tractographic representation of structural connectivity maps (connectomes, projectomes) based on single-cell (neurons) data^64–68^ or to analyze the distribution patterns of the microvasculature and of the brain-wide arterial and venous cerebral vascular system, particularly in relation to the different brain regions. In tumors differences in the distributions between different cell populations (e.g. tumor-associated macrophages and cancer cells) and their relation with respect to the vasculature, collagen or drug distributions could be also obtained, providing a better understanding of the whole tumor microenvironment.

The imaging scheme could be also used as an invaluable quantitative tool to independently validate local tissue structures derived from diffusion magnetic resonance images^48,57,69–72^ and to relate pathology with DTI information^57^.

The flexibility of our approach ensures it is translatable to other imaging techniques. This is an important feature because it could allow to utilize data collected with different imaging techniques and based on different contrast mechanisms, such as for example optical coherence tomography, micro-XCT, synchrotron radiation phase contrast imaging, and histopathology. A multimodal approach based on the combination of two or more of the aforementioned techniques may also provide synergistic and complementary information, offering the possibility to overcome limitations present in the single modalities. We have focused on fixed image samples, but our approach could be also applied in *in vivo* settings, for the characterization for example of cell migration in tumors.

Finally, we emphasize that our method is highly valuable for seamlessly studying data on micro, meso- and macroscale organization significantly improving abstraction. These goals are difficult to achieve by conventional microscopic or macroscopic imaging technologies alone; rather, potent processing methods such as the one here illustrated are needed to distill the most relevant information from large-scale datasets.

## Materials and Methods

### Animals and experimental parameters

C57BL/6 (000664) mice were purchased from Jackson Laboratory (Bar Harbor, ME, USA) and maintained in our pathogen-free environment at the Massachusetts General Hospital animal facility (MGH, Boston, MA, USA). Animal experiments were performed in compliance with institutional guidelines and approved by the Subcommittee on Animal Research Care at Massachusetts General Hospital. Eight-week-old mice were used for experiments. For *in vivo* microvasculature labeling, mice were anesthetized with 1.5–2% isoflurane supplemented with oxygen prior to intravenous injection of Rhodamine-labelled Griffonia simplicifolia lectin (100 µl; RL-1102, Vector Laboratories, Burlingame, CA, USA). Mice were sacrificed five minutes after *in vivo* labeling of endothelial cells and perfused through the left ventricle with 20 mL of ice-cold PBS followed by 20 mL of 4% formaldehyde solution in PBS (Thermo Scientific, Waltham, MA, USA). Hearts were harvested, post-fixed for an additional hour at room temperature, washed in PBS, embedded in 4% agarose and cut in 300 μm sections using a Pelco 101 vibratome.

### Optical clearing

Heart tissue section clearing was performed using a slightly modified version of the CUBIC (clear, unobstructed brain imaging cocktails and computational analysis) method^14^ for multicolour imaging of fluorescent proteins and/or immunostained samples. The method is based on immersing fixed tissue slices in a chemical mixture containing aminoalcohols. After sectioning, tissue slices were immersed in a 30% glycerol solution for 1 h and stored at −80C. Prior to imaging, slices were brought back to room temperature, washed twice in PBS for 10 minutes and immersed for 30 min in a solution obtained by mixing 25 wt% urea (U16–3, Fisher Scientific Hampton, NH, USA), 25 wt% N,N,N0,N0-tetrakis(2-hydroxypropyl) ethylenediamine (50-014-48142, Fisher Scientific) and 15 wt% Triton X-100 (85111, Life Technologies, Carlsbad, CA, USA). Slices were then mounted in a custom-made imaging holder and allowed to mechanically relax for 30 minutes prior to imaging.

### Confocal microscopy

Images at 1.2 microns/pixel planar resolution (512 × 512) were acquired (integration time, 2.0 microsecond/pixel) as a function of depth (z-stack, 4 μm step size) using a customized Olympus FV1000 system based on a BX61-WI confocal microscope (Olympus America).

A XLUMPLFLN 20x water immersion (NA 1.0, Olympus America, 2 mm WD), a XLUMPlanFl 10x water immersion (NA 0.6, Olympus America, 2 mm WD) and a UPLSAPO 30x silicon immersion (NA 1.05, Olympus America, 0.8 mm WD) were the objectives used for data collection.

### Imaging probes

Lectin was imaged using a 559 nm diode lasers, respectively, in combination with a dichroic beam splitter (DM405/488/559/635 nm). Fluorescence was collected using appropriate combinations of beam splitters (SDM560) and emission filters (BA575–620).

Rhodamine-labelled Griffonia simplicifolia lectin (RL-1102, Vector Laboratories) was used as an intravital stain to outline the microvasculature. The dye-labelled lectin (lectin-TRITC) has a 550 nm maximum excitation and a 575 nm maximum emission and was excited using a 559 nm diode laser. Fluorescence was collected using an appropriate combination of beam splitters (SDM560) and emission filters (BA575–620).

### Data analysis

Analyzing data and coordinate transformations among different visualization datasets was performed using Matlab (The Math Works, Natick, MA, USA) in integration with Camino, Diffusion Toolkit, Trackvis, Paraview, ITKsnap and Amira (FEI). Data elaboration was performed on several platforms. Image processing was performed on a Precision Tower Workstation Intel Xeon Processor E5-2637, 3.5 GHz, 64 bit, equipped with an NVIDIA GeForce GTX 1080 graphics card, 128 GB RAM, a fast-drive PCI 512 GB SSD Samsung 950 Pro, running Windows 10. Codes for the 3D segmentation were written using Matlab, Python (Python Software Foundation, Wilmington, DE, USA), and Tensorflow. Matlab-based multilayer feed-forward neural networks training was performed locally on a GTX 1080 graphic card. Tensorflow-based CNN training was performed on the ERIS One computing cluster utilizing two Tesla P100 Nvidia GPUs. Skeletonization and graph analysis were performed on 10 virtual machines instances on Azure (D2s-64 series, 128 GB RAM).

### Image processing

Microscopy images were image processed in order to increase both SNR and contrast ratio. Images were first de-noised using a BM3D filter method. Data were then interpolated along axial direction and deconvolved to sharpen images. Deconvolution and de-noise were performed on a local GPU (GTX 1080) to increase performance (approximately a factor 100 acceleration) (Supplementary Fig. S2).

### Data segmentation

After image processing (Supplementary Fig. S2), images were segmented to extract underlying information and then post-processed to remove segmentation-related artifacts. After exploring some previously published techniques^2^, we decided to develop a new custom-built solution based on machine learning. This approach has several advantages: our measurements generate a great deal of data, which is a key requirement for the training stage when using complex learning models; new datasets may further improve the classifier; suitable artificial intelligent development programming platforms and online resources are readily available. While we have here adopted a custom-based solution based on machine learning, we note that the specific algorithmic route chosen for producing the segmented data can be different depending on the dataset complexity as well as the origin of the signal contrast (e.g. fluorescence, absorption, scattering, X-CT, synchrotron radiation, etc.) or other personal preferences. To that aim different approaches are possible and recommended, and they all represent the preliminary step to obtain data binarization, which is necessary for extracting the sets of morphological descriptors from which volumetric tensor-value representation can be generated.

Specifically we chose the supervised learning paradigm to develop specific neural networks for our project. Because biological structures are three-dimensional, we selected volumetric approaches, which also address our imaging protocol’s anisotropic resolution, with a slight elongation along the vertical axis. To train networks, we used manually annotated datasets as training, validation and test datasets. While several frameworks are available for deep learning, we focused on Matlab and Tensorflow for, respectively, multilayer feed-forward neural networks and convolutional neural networks (CNN).

In Matlab, we used training datasets (6) to train a multilayer feed-forward neural network consisting of four-hidden layers and seven neurons each (Supplementary Fig. S3). The input was a 3D cubic stack patch of 7 × 7 × 7 normalized data concatenated to a raw data subset to allow the network to learn the sample features while also considering signal intensity. The network was designed to classify the 3D stack’s central point as belonging or not to a certain biological structure. The network output was thresholded to assign a designated class. Network performances were computed through the Dice coefficient calculated on the datasets (values up 0.8, for appropriate threshold).

The average Dice coefficient calculated on independent datasets (n = 11) extracted within a short axis slice (Supplementary Fig. S4A) is equal to 0.85.

While the above network is fast, has simple architecture and can be used with local hardware resources, we also investigated a more complex network based on Tensorflow, which can work on remotely connected computational platforms. In Tensorflow, the structure of the 3D Convolutional Neural Network (CNN) was adapted from the V-Net and retains the 29 convolutional layers present in the original, with some adaptations. The number of filters (5 × 5 × 5) learned in the first layer is 24 and doubles every down-convolution. Down-convolution is performed using a volume of 2 × 2 × 2 and stride 2. All activation functions used prior to the final layer are Rectified Linear Units. All variable initializations for all weights used the Xavier initialization and all variable initializations for the biases used zero initialization. A batch normalization layer was added to each set of layers before a down-convolution was performed. This network was implemented in TensorFlow 1.7, and all code was written in Python. Training was performed on the ERIS One computing cluster utilizing two Tesla P100 Nvidia GPUs for training and forward passes of the network.

Our 3D CNN was trained on five manually annotated training volumes. The size of the volumes was reflectively padded along *XY* to the size of 352 × 352 × *Z*. Here the depth *Z* varied in size between 200–400 slices. The batch size consisted of only one training example. From each of the five volumes, the network was given an input slice stack of image data, size 352 × 352 × 5, and a slice of segmented label, size 352 × 352 × 1. The segmented label corresponded to the middle of the input image slice stack. The network’s output volume matched the segmented data size. After one batch had been used for trained, the input data and segmented label shifted down one slice. Training followed this pattern until the entire training volume stack had been processed, and training on all five volumes was considered one epoch. The network was trained by reducing the cross-entropy loss between the network output and the expert-obtained segmented labels. ADAM was used as the optimization algorithm.

The network was monitored during each epoch by checking the performance on one volume of previously unseen validation data using the Dice coefficient loss. Since the network’s output layer is a softmax layer, a naïve threshold of 0.5 was used to test against the binary values in the segmented labels. The network model was saved once convergence was determined by the network designer. After convergence, the model received a Dice coefficient test score of 0.81 on another previously unseen test volume. Despite their differences, the two NN architectures produced very similar results. We decided therefore to elaborate all the data in a local Matlab framework for convenience.

The direct comparison between the 3D rendering of the fluorescence imaging data (Fig. 1a) and the thresholded network output (Fig. 1b) demonstrates the high degree of overlap present between the two datasets, as suggested by the Dice coefficient.

### Expert-annotated datasets

We took particular care to obtain excellent annotated datasets. We built a total of eight annotated datasets for training, validation and testing. Typical sizes are in the range of 300 × 300 × 250 microns. Randomly selected areas were chosen within a mid short-axis heart slice (300 microns thickness). For each area, two image datasets were acquired. One dataset was acquired using the same experimental acquisition condition used for whole heart imaging, including the post-acquisition image processing pipeline (Supplementary Fig. S1). The other dataset was obtained while imaging the same region but using an oil-immersion objective to better match the refractive index of the clearing solution and therefore reduce the axial point spread function. Also, imaging acquisition conditions were changed in order to maximize SNR, thereby increasing the laser power and integration time while reducing PMT gain. This was done in order to facilitate the microvasculature segmentation and was performed by an expert annotator using local orthogonal sections to exploit the volumetric information and enhance structural recognition.

In order to achieve spatial matching between the two datasets, it was necessary to ensure the samples did not move when objectives were exchanged. We also conducted automatic registration based on cross-correlation between the two datasets. After being manually inspected, the registered datasets were properly cropped to trim borders and match the datasets. The microvasculature segmentation obtained from the oil-based acquisition was then used as a guideline to facilitate manual annotation of the water-based objective-acquired dataset. This annotation was performed using local orthogonal sections for structural recognition enhancement. These segmentations were considered our “gold standard”.

### Skeletonization

The binary segmentation data were skeletonized using a 3D medial surface/axis thinning-based algorithm by Lee *et al*. The algorithm finds the centerline of the binarized vessels through a fast, iterative erosion process, using Skeletonize3D on an existing Fiji/ImageJ plugin. Quantitative analysis of the skeletonized images is performed using AnalyzeSkeleton, another Fiji/ImageJ plugin, to obtain a direct graph representation that includes the coordinates of all nodes and skeleton branches and classifies voxels based on their number of neighbors: end-points (<2), junctions (>2) or slabs (=2).

Figure 1c shows the high degree of accuracy achieved in identifying the skeletonized microvasculature throughout the dataset represented in Fig. 1a. This is emphasized also in Supplementary Movie 7, where an axial view of the microvasculature fluorescence signal is represented as it moves along the vertical direction. The 3D rendering of the skeletonized microvasculature was overlaid to better follow the identification of the microvasculature tracts and branches.

### Vector and tensor fields

Tensor fields were obtained by extracting information contents from the segmented images and then computing using a sliding window. All biological structures within the window were transformed into feature descriptors (in the microvasculature, segments represent adjacent nodes connections) and centered on their midpoint. All extreme points of the grouped segments were used as a points cloud to estimate the dispersion through a tensor (variance-covariance matrix). While an arbitrary value can be chosen, we chose a mesoscale range typical of techniques such as MRI. The window size was set at 90 × 90 × 90 microns, while the window shifting spacing was equal to 20 microns. The vector fields were obtained by selecting the principal eigenvector from each coordinate point in the tensor field. Vector and tensor maps were weighted by the number of elements present within each single volume (Supplementary Fig. S8g) in order to attribute statistical significance to the voxels. Large window sizes are preferred to obtain smoothed vector/tensor fields.

### Tractography

Eigenvector tracking was performed along the three principal eigenvectors using Diffusion Toolkit and Trackvis. Tracks were generated from all voxels using a tracking deterministic method based on the 2nd order Runge-Kutta technique. A propagation angle of greater than 35 degrees was used as single termination criterion. No FA thresholds were implemented. Typically, only 15% of the tracks are displayed. This angle is substantially higher than the propagation angle of normal myofiber tracts, which is <4o per voxel at mesoscopic resolution, but is required for complete resolution of a branching continuum such as a vascular tree. No FA thresholds were implemented. Typically, only 15% of the tracks are displayed. Tractography of myofiber tracts by MRI in the murine heart (Supplementary Fig. S11g) was performed with diffusion spectrum imaging as previously described^20^.

### Ethical approval

All animal procedures and protocols were approved by the Institutional Animal Care and Use Committee of the Massachusetts General Hospital, and they are in accordance with the NIH Guide for the Care and Use of Laboratory Animals.

## Supplementary information

Supplementary Information.

Supplementary Movie 1.

Supplementary Movie 2.

Supplementary Movie 3.

Supplementary Movie 4.

Supplementary Movie 5.

Supplementary Movie 6.

Supplementary Movie 7.

Supplementary Movie 8.

Supplementary Movie 9.

Supplementary Movie 10.

Supplementary Movie 11.

Supplementary Movie 12.

Supplementary Movie 13.

Supplementary Movie 14.

Supplementary Movie 15.

Supplementary Movie 16.

## Data Availability

The deep learning model and the Matlab-based multilayer feed-forward neural networks reported in this work use standard libraries and scripts that are publicly available in both TensorFlow and Matlab. The raw images training datasets for the experiments are available from the corresponding author upon request. The trained networks are also available upon request.
