# Supplementary material for: Fluorescence microscopy tensor imaging representations for large-scale dataset analysis
Source: Sci Rep. 2020 Mar 27;10:5632. doi: 10.1038/s41598-020-62233-2 (PMC7101442; doi:10.1038/s41598-020-62233-2)
Supplement: Supplementary file 1 — Supplementary Information. [file 41598_2020_62233_MOESM1_ESM.docx]

**Supplementary Information**

**Fluorescence microscopy tensor imaging representations for large-scale dataset analysis.**

Claudio Vinegoni,^1,*,†^ Paolo Fumene Feruglio,^1,2,†^ Gabriel Courties,^1^ Stephen Schmidt,^1^ Maarten Hulsmans,^1^ Sungon Lee,^3^ Rui Wang,^4,5^ David Sosnovik,^6,7^ Matthias Nahrendorf,^1,8^ and Ralph Weissleder^1,9^

**SUPPLEMENTARY MOVIES**

**Movie 1.** 3D renderings of the automatically segmented vasculature and XY ortho-plane view on the corresponding fluorescence signal (Vol. 1 Fig. S4).

**Movie 2.** 3D renderings of the automatically segmented vasculature and XZ ortho-plane view on the corresponding fluorescence signal (Vol. 1 Fig. S4).

**Movie 3.** 3D renderings of the automatically segmented vasculature and YZ ortho-plane view on the corresponding fluorescence signal (Vol. 1 Fig. S4).

**Movie 4.** 3D renderings of the automatically segmented vasculature and XY ortho-plane view on the corresponding fluorescence signal (Vol. 2 Fig. S4).

**Movie 5.** 3D renderings of the automatically segmented vasculature and XZ ortho-plane view on the corresponding fluorescence signal (Vol. 2 Fig. S4).

**Movie 6.** 3D renderings of the automatically segmented vasculature and YZ ortho-plane view on the corresponding fluorescence signal (Vol. 2 Fig. S4).

**Movie 7.** 3D renderings of a representative skeletonized volume of a lectin-TRITC-stained heart (compare with Fig. 1c).

**Movie 8.** 3D rendering of the fluorescence microvasculature signal in a representative short-axis basal slice (compare with Fig. 1e).

**Movie 9.** 3D rendering of a representative volume of lectin-TRITC-stained microvasculature and corresponding 3D tractogram (compare with Fig. 2a, 3j).

**Movie 10.** 3D renderings of a representative volume of lectin-TRITC-stained microvasculature and corresponding skeletonized and glyphs representations (compare with Fig. 3m, n and Fig. 2i).

**Movie 11.** 3D renderings of the tomographic vector field representation of the vascular directional primary eigenvector in a representative apical short-axis slice (compare with Fig. 4a) and relative 3D tractogram.

**Movie 12.** 3D renderings of the microvasculature in a representative apical short-axis slice and relative 3D tractogram (compare with Fig. 4g, b).

**Movie 13.** 3D renderings of the microvasculature in a representative basal short-axis slice and relative 3D tractogram.

**Movie 14**. 3D renderings of the microvasculature in a representative sagittal long-axis slice and relative 3D tractogram (compare with Fig. 4p,q).

**Movie 15**. 3D renderings of macrophage distribution in a Cx3cr1GFP/+ mouse, within a sagittal long-axis slice (compare with Fig. 5a).

**Movie 16**.3D tractogram rendering of macrophage distribution in a Cx3cr1GFP/+ mouse, within a sagittal long-axis slice (compare with Fig. 5f).

**SUPPLEMENTARY FIGURES**


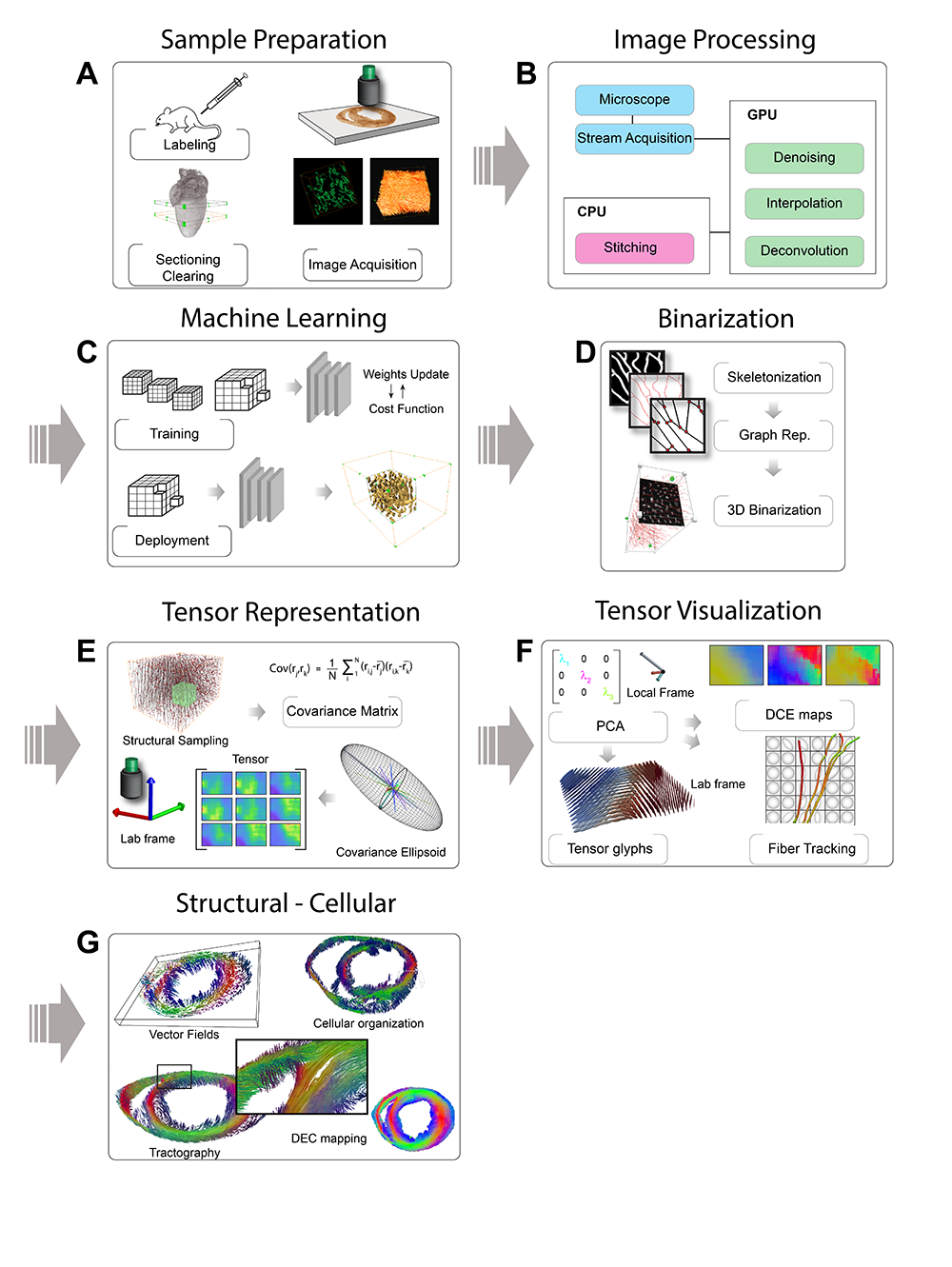


**Figure S1.** Schematic overview of the general acquisition and processing scheme. (a) Sample preparation and acquisition phase. (b) Datasets are image processed for increased SNR and contrast ratio. (c) Machine learning algorithms perform automatically-based cellular or vascular segmentation. (d) Data are binarized using a skeletonization algorithm and graph representations are created. (e) Fluorescence microscopy tensor imaging representations are generated through covariance matrices. (f) Tensor visualization maps and fiber tracks are obtained from volumetric tensor-valued representations (g). Structural and cellular maps give an overview of the underlying structural order.


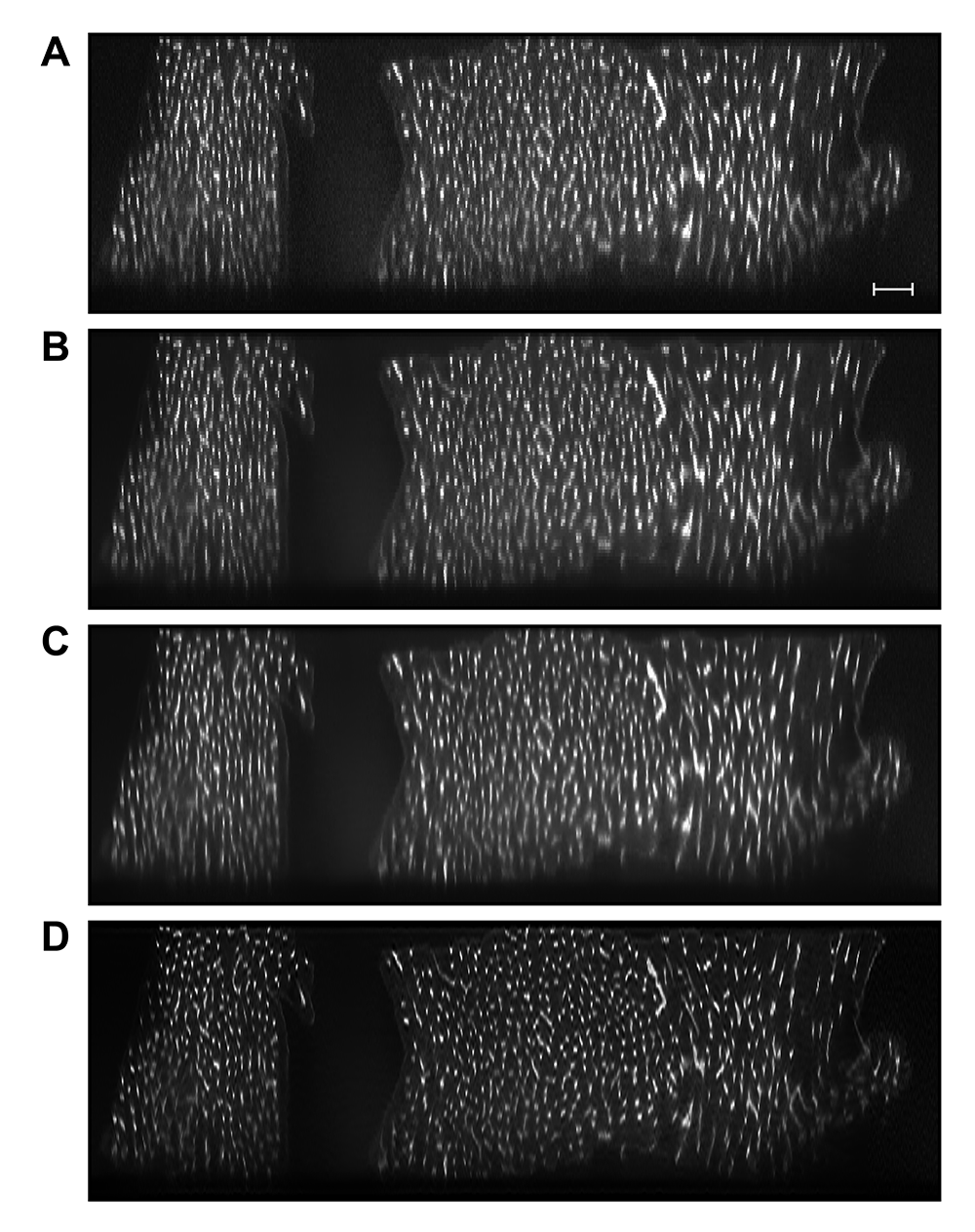


**Figure S2.** Microscopy data are image processed in order to increase SNR and contrast ratio (see Methods). Raw data (A) are first denoised using a BM3D filter (B). Data are then interpolated along the axial direction (C) and finally deconvolved for increased sharpening (D). Deconvolution and denoise code are run on a local GPU for increased performance (approximately a factor 100 acceleration). Scale bars, 50 μm.


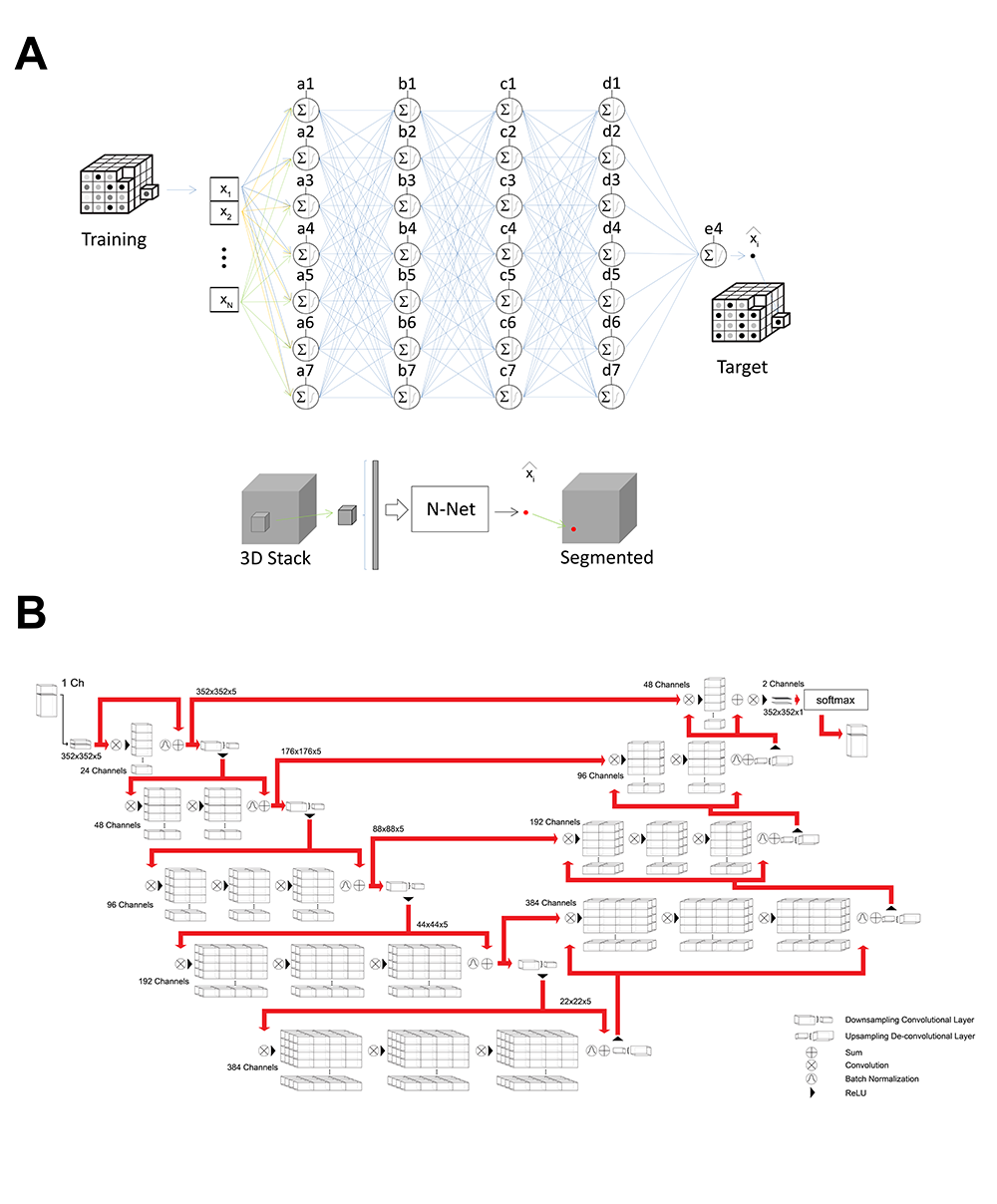


**Figure S3.** (A) Scheme of the neural network architecture used for automatic microvasculature segmentation (see Methods). Manually segmented training datasets were divided into volumetric cubic blocks and used to compute the weights (network lines) and bias parameters (ai, bi, ci, di and e). The input size, a 7x7x7 normalized-valued array concatenated to a subset of the raw values within the cube, allowed the network to adjust its parameters based on both geometrical features and intensity of the original signal. The binary output xi is the central voxel of the cube extracted from the manually annotated training dataset. In this implementation, except for the output, the other 4 layers count 7 neurons each. Input, neurons and layer size can all be easily increased at the expense of computational time for both training and network feeding. Segmentation is performed by feeding the network (N-NET) with the acquired data, organized in subsets (3D stacks) as the training inputs. The output is then obtained by running the network with the computed weights and biases calculated in the training step. All subsets are then rearranged as in the raw dataset and finally thresholded for binarization. (B) Structure of the 3D Convolutional Neural Network (CNN) used to segment the blood vessels (see Methods). This network combines 29 convolutional layers. The input shape is a stack of 352x352x5 pixels. The output shape is a single slice, 352x352x1. The left side of the network correspond to the contracting/down sampling path; the input size reduces by half while the number of channels doubles. The right side of the network corresponds to the expanding/upsampling path; the input size doubles and the number of channels is reduced by half. At the end of each layer, before either an up-sampling or down-sampling convolution, a batch normalization operation is performed. Each convolution includes a rectified linear unit (ReLU) to provide non-linearity to the network. The network also contains feed-forward features from the left to the right side.


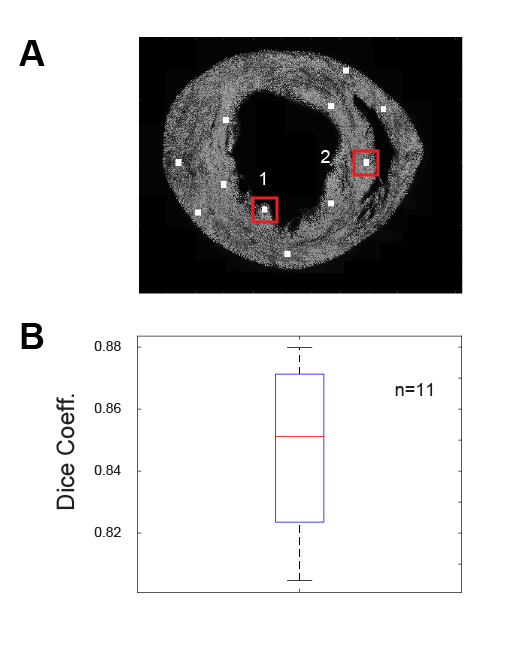


**Figure S4.** Dice coefficients on selected volumes within an optical short-axis slice. A) 11 selected volumes B) Dice coefficients for the volumes in A (n=11).


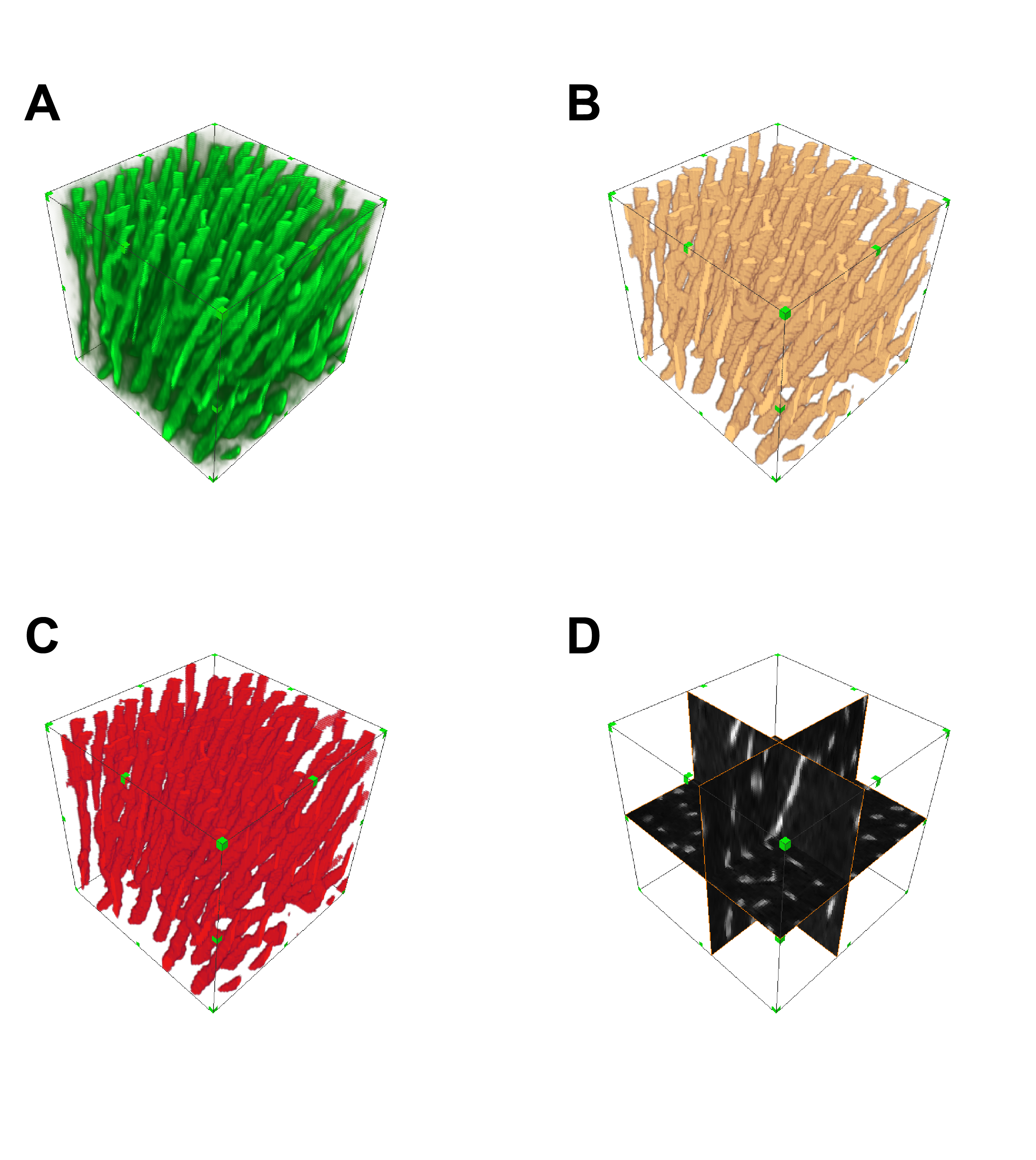


**Figure S5.** 3D renderings of the vasculature corresponding to the Vol. 1 in Figure S4. A) Fluorescence signal. B) Manual segmentation. C) Automatic segmentation. D) Ortho-view of the fluorescence signal.


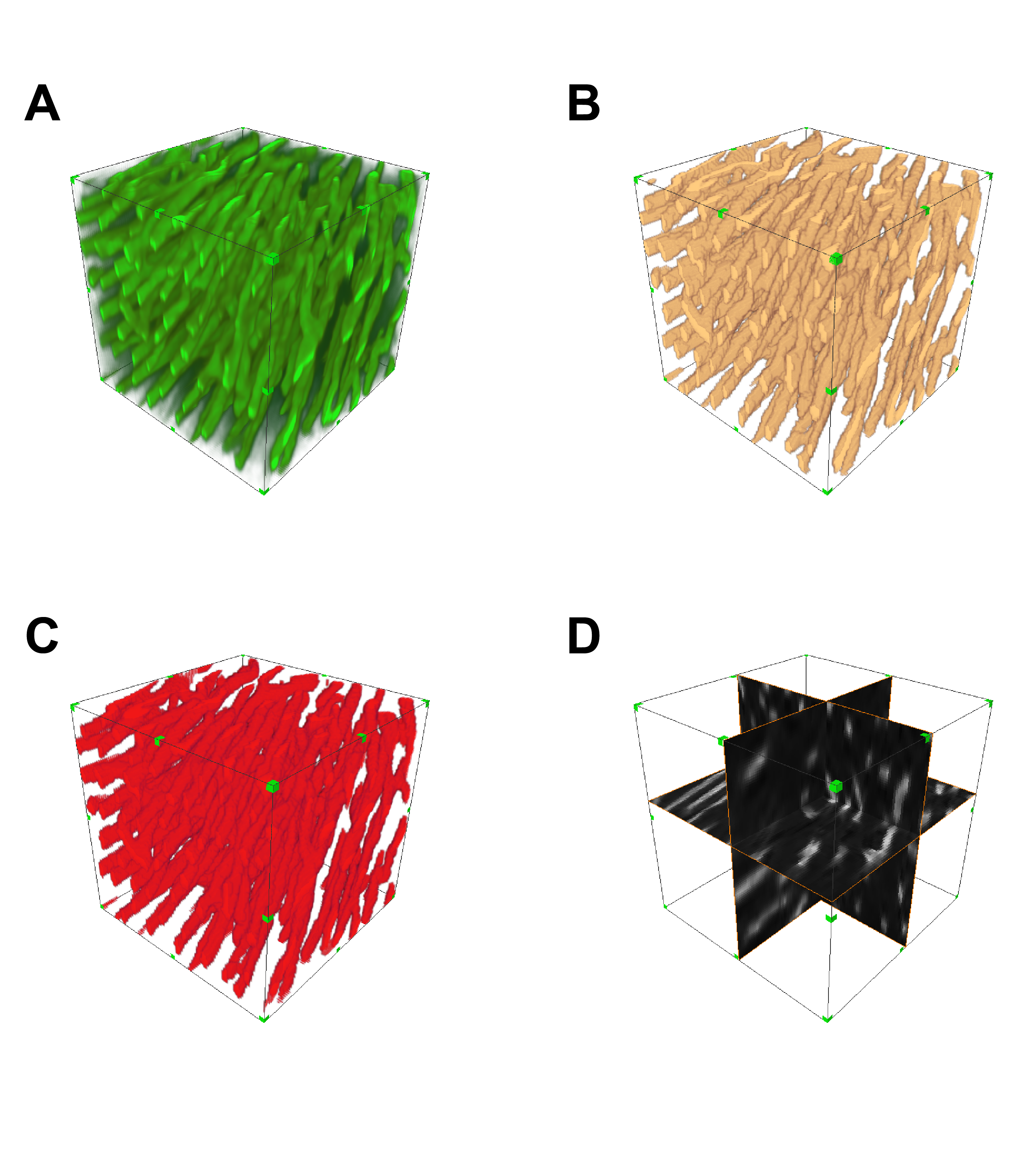


**Figure S6.** 3D renderings of the vasculature corresponding to the Vol. 2 in Figure S4. A) Fluorescence signal. B) Manual segmentation. C) Automatic segmentation. D) Ortho-view of the fluorescence signal.


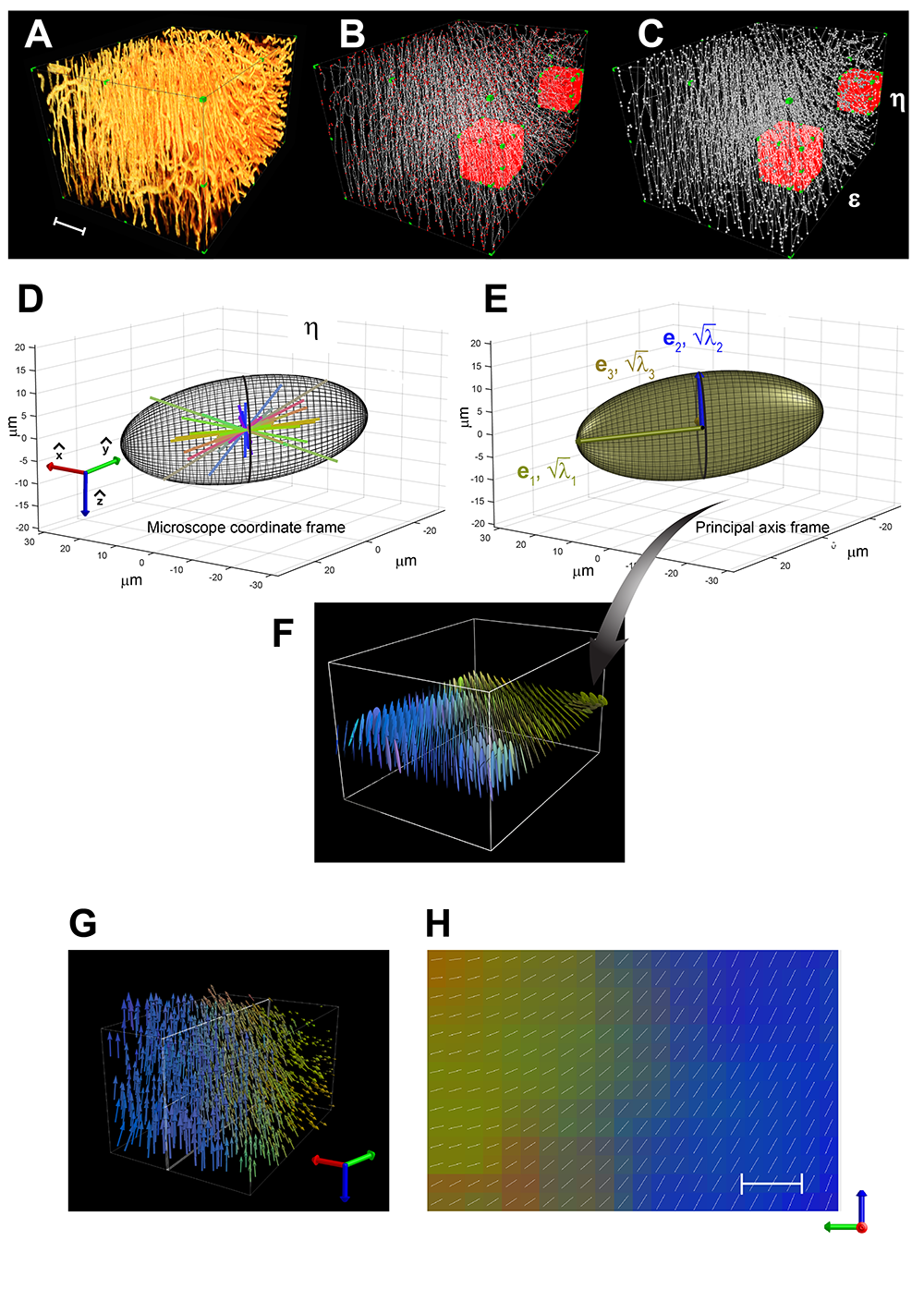


**Figure S7.** (A) 3D renderings of a representative volume of lectin-TRITC-stained cardiac microvasculature as in Fig. 1. (A) Image processed fluorescence data, (B) corresponding skeleton and graph nodes, (C) nodes and their connecting segments. Bounding box, 250x205x340 μm. (D) 3D representation of the covariance ellipsoid for the microvasculature network present within the sampling volume η. (E) The principal components v1, v2, v3 give the main vascular directionality within the sampling volume η. (F) 3D ellipsoid glyph-based visualization. (G) Sagittal cut view of the plane represented in Fig. 3d with corresponding direction color encoded (DEC) map of the principal eigenvector v1 (H). RGB components are defined as the principal eigenvector’s absolute XYZ values. Scale bars, 50 μm.


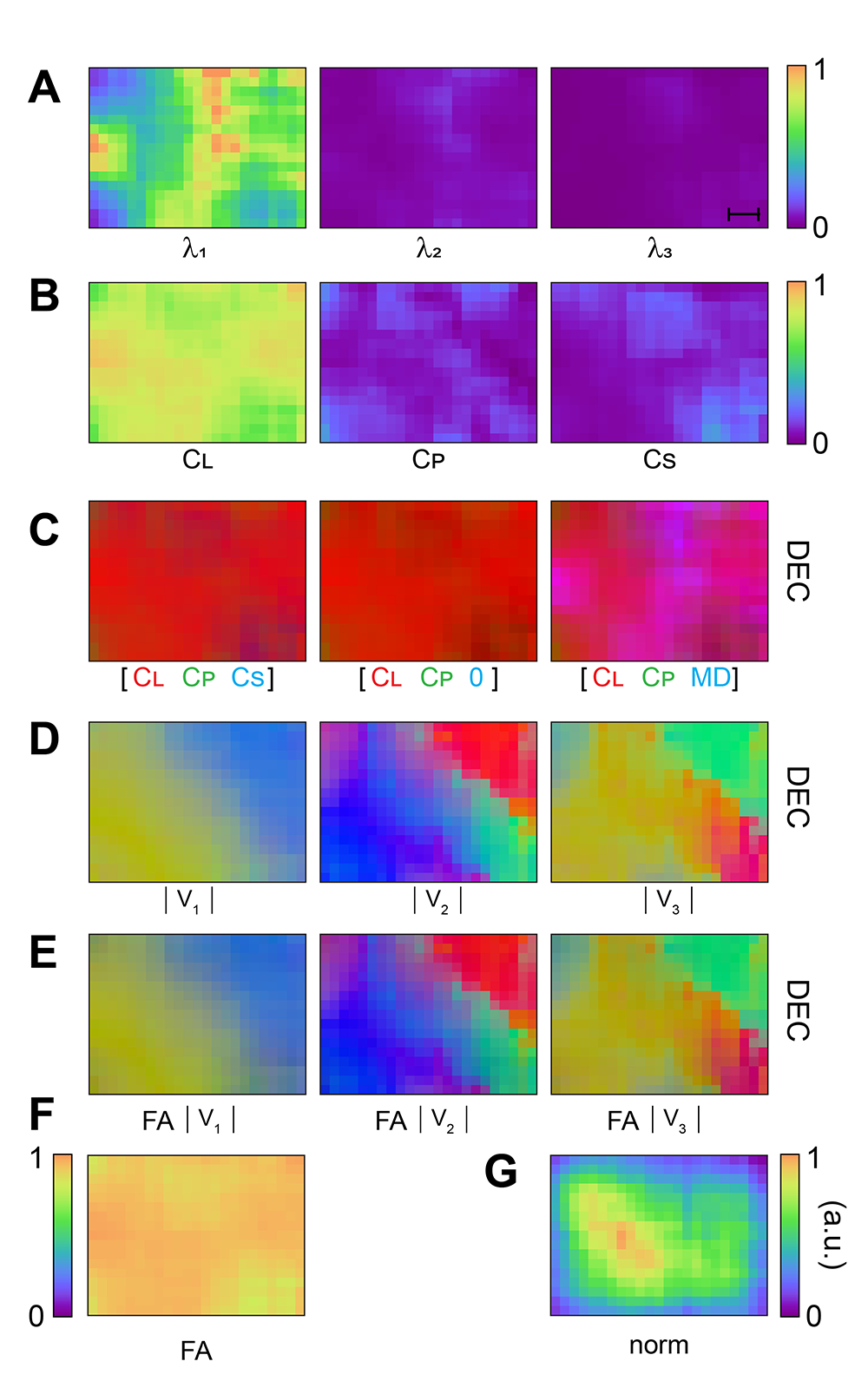


**Figure S8.** Two-dimensional maps to visualize tensor metrics. (A) Three sorted eigenvalue maps (λ1, λ2, λ3), and (B) Westin-measures cl, cp, cs with relative pseudo-color map. (C) RGB color-coded map obtained combining Westin-measures with different metrics. (D) Direction color encoded (DCE) maps of the principal eigenvectors v1, v2, v3 and their corresponding fractional anisotropy (FA)-weighted representations (E). The RGB components are defined as the eigenvector’s absolute XYZ values. (F) Fractional anisotropy and (G) normalization density map as obtained by counting the number of nodes present within each voxel. Scale bar, 50 μm.


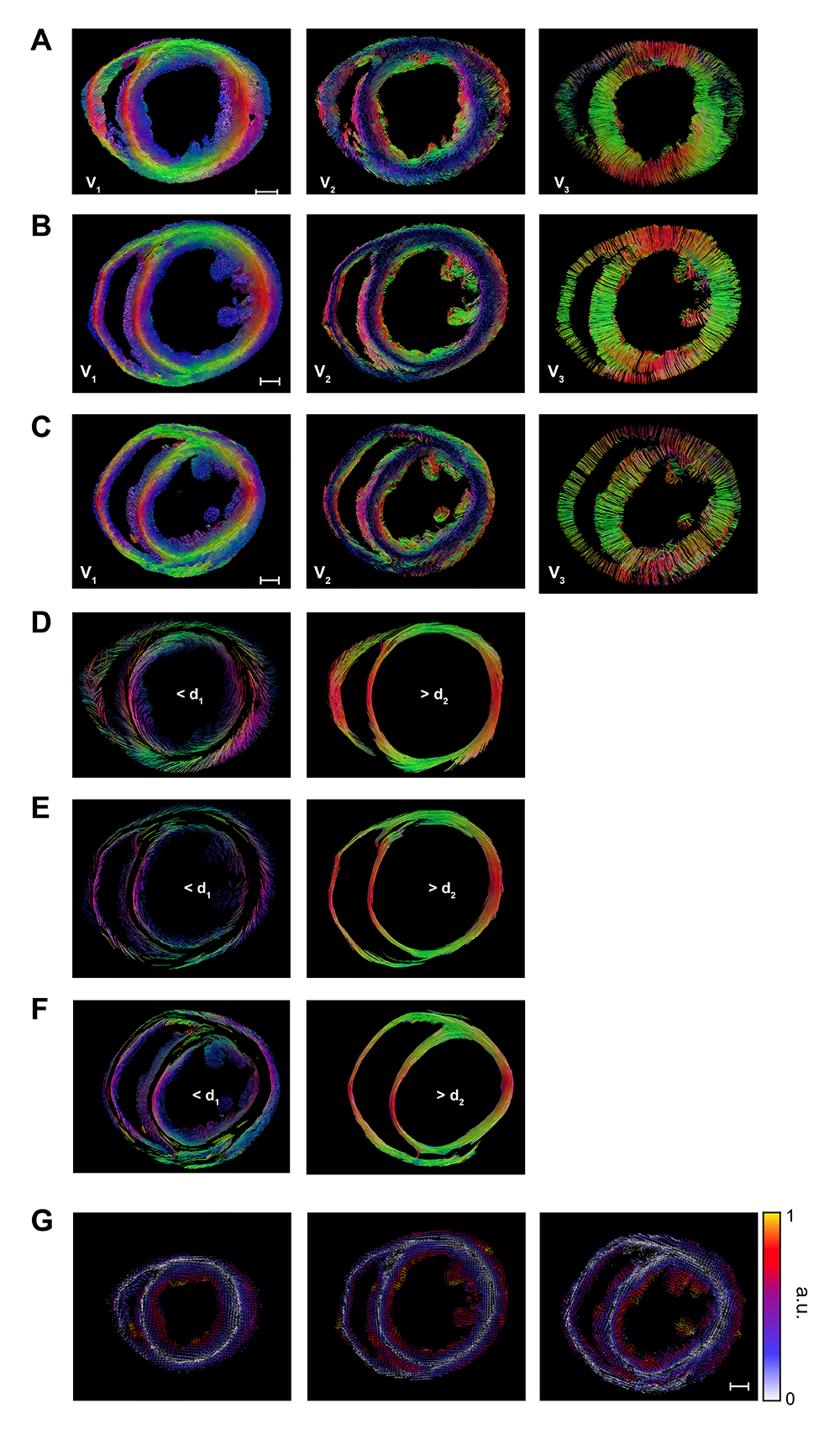


**Figure S9.** Tomographic vascular tractograms determined along the primary (left), secondary (mid) and tertiary (right) eigenvectors for a representative base (A), mid (B) and apical (C) short-axis heart slice. (D-F) Tractograms for the respective heart slices, with fiber lengths smaller than 600 μm (left side) and larger than 1000 μm (right side). (G) 2D maps of the principal eigenvector’s projection along the vertical axis for a base, mid and apical short-axis heart slice. Scale bars, 500 μm.


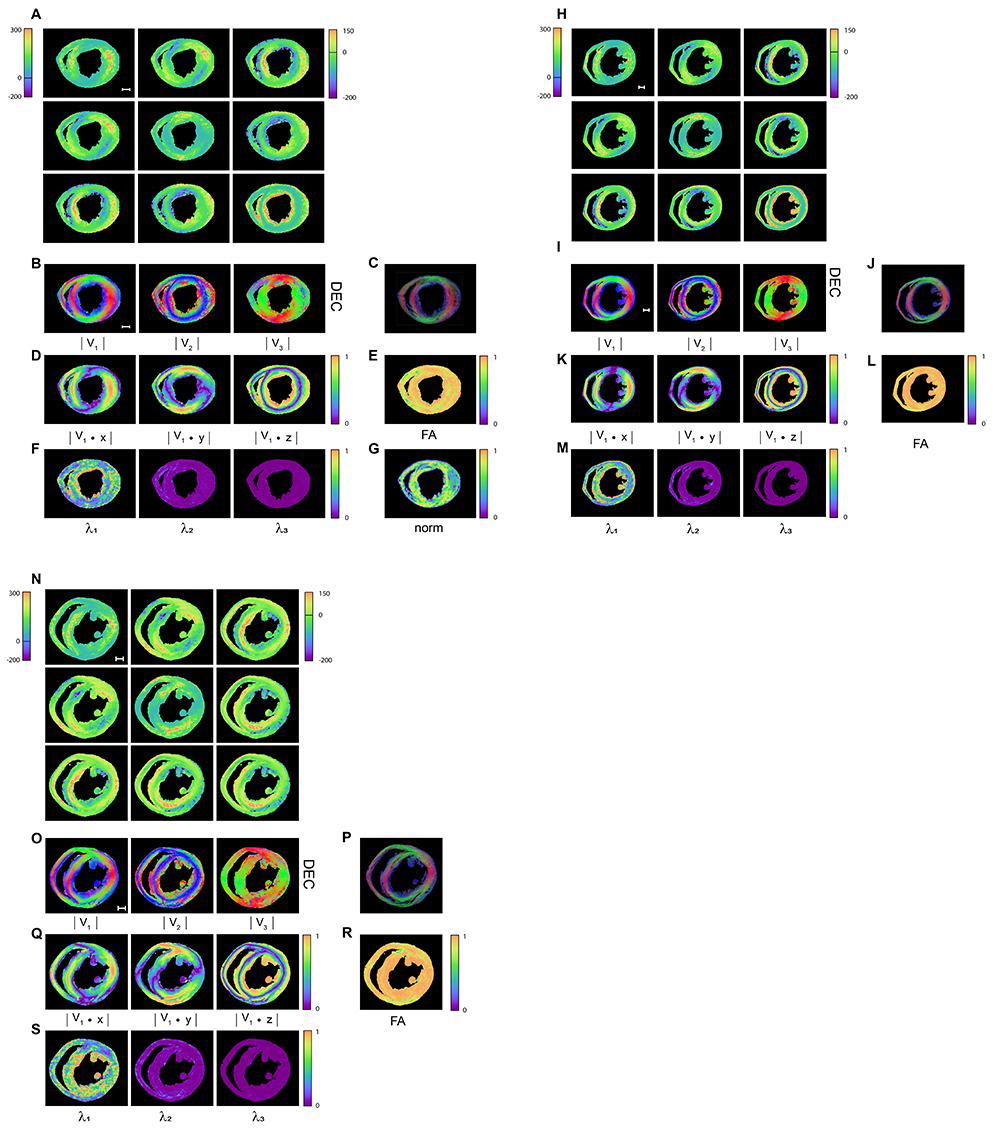


**Figure S10.** Microvascular distribution in an apical (A-G) short-axis heart slice. (A) Image representation of the tensor components, using a pseudo color map. Off diagonal components are rescaled for better contrast. (B) Direction color encoded (DCE) maps of the principal eigenvectors v1, v2, v3. The RGB components are defined as the absolute values of the XYZ components of the eigenvector. (C) Direction color encoded skeleton axial view representation of the vascular network. (D) Maps of the principal vector components along the lab frame. (E) Fractional anisotropy map. (F) Maps of the three sorted eigenvalues maps (λ1, λ2, λ3). (G) Normalization density map as obtained by counting the number of nodes present within each voxel. Scale bar, 500 μm. Microvascular distribution in a mid short-axis heart slice (H-M). (H) Image representation of the tensor field components, using a pseudo color map. Off diagonal components are rescaled for better contrast. (I) Direction color encoded (DCE) maps of the principal eigenvectors v1, v2, v3. The RGB components are defined as the absolute values of the eigenvector’s XYZ components. (J) Direction color encoded skeleton axial view representation of the vascular network. (K) Maps of the principal vector components along the lab frame. (L) Fractional anisotropy map. (M) Maps of the three sorted eigenvalues maps (λ1, λ2, λ3). Scale bar, 500 μm. Microvascular distribution in a basal short-axis heart slice (N-S). (N) Image representation of the tensor components, using a pseudo color map. Off diagonal components are rescaled for better contrast. (O) Direction color encoded (DCE) maps of the principal eigenvectors v1, v2, v3.The RGB components are defined as the absolute values of the eigenvector’s XYZ components. (P) Direction color encoded skeleton axial view representation of the vascular network. (Q) Maps of the principal vector components along the lab frame. (R) Fractional anisotropy map. (S) Maps of the three sorted eigenvalues maps (λ1, λ2, λ3). Scale bar, 500 μm


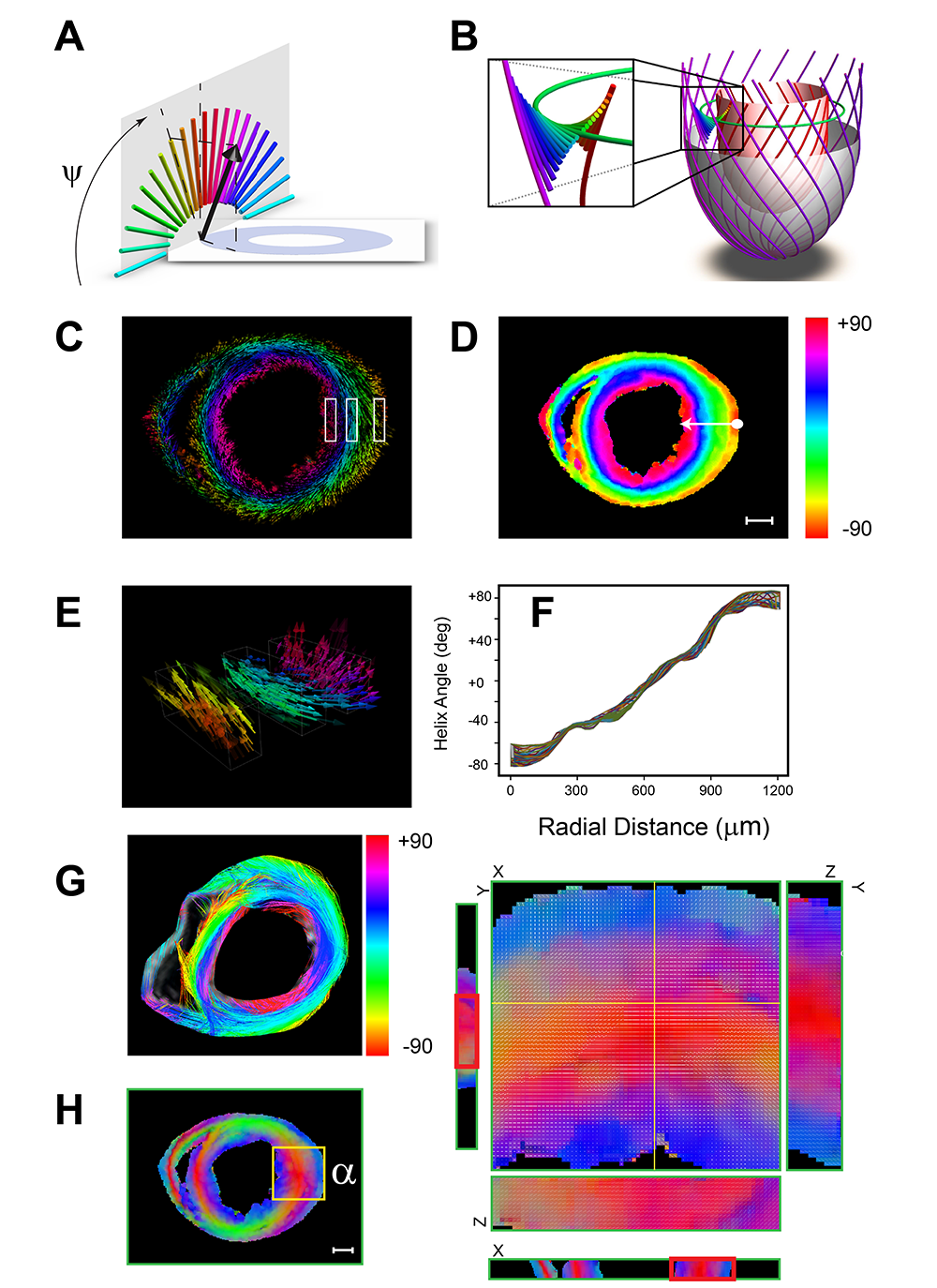


**Figure S11.** Defining a new set of coordinate system relative to the local wall better visualizes and quantifies the cardiovascular orientation (A). Here the eigenvectors’ orientation is defined by two quantities, the helix and the transverse angles. The helix angle () is defined as the angle of the primary eigenvector, indicating the vascular direction with the horizontal plane parallel to the epicardial tangent plane. The projection of the primary eigenvector on the tangent plane is colored according to the value of the helix angle. Schematic view (B) illustrating the characteristic myocyte orientation in the LV of a whole heart, with color-coded emphasis on transmural change in the helix angle (zoomed area) [88]. The myocyte fibers are oriented parallel to the epicardial tangent plane with radially oriented sheets, displayed in the figures as round cylinders along the directions of the primary eigenvector.

A representative 3D primary eigenvector field visualization (C) and 2D helix map (D) of a single cardiac slice. In the epicardium and endocardium area, the primary eigenvectors are mostly oriented in the longitudinal apex-base direction. In the mid-wall, the fibers are oriented predominantly in the circumferential direction. At each voxel, the helix angle value of the primary eigenvector is displayed in different colors according to a specific color map. In particular, a circular HSV color map was chosen to connect angles at +90 and -90 degrees. The variation in color emphasizes the primary eigenvector’s epi-to-endocardial counterclock-wise helix angular rotation (from about – 80 to +80 degrees), as better seen in the zoomed area (E). The helix angle’s transmural change can be seen in greater detail in (F), where the helix angle is plotted along the transmural depth from epi- to endocardium. The direction occurs along the white arrow indicated in (D).

The orientation of the vascular network gradually rotates counterclockwise, similar to the classical myocardial fiber orientation, from the epicardium to the endocardium [89]. This orientation gives rise to a local microvascular helical architecture with a transmural angle gradient of roughly 150°. (G) A 3D tractogram color coded for HA content as obtained by diffusion spectrum-MRI (DSI). (H) Directionally encoded color map of the principal eigenvector. (I) Magnified DEC map of the region labeled α in (H), with overlayed hedgehogs, representing the length of the primary eigenvector projection along the vertical axis. The shorter the hedgehog representation, the steeper the fiber inclination. The hedgehogs show that the mid-wall primary eigenvectors are oriented predominantly in the circumferential direction. Scale bar, 500 μm.


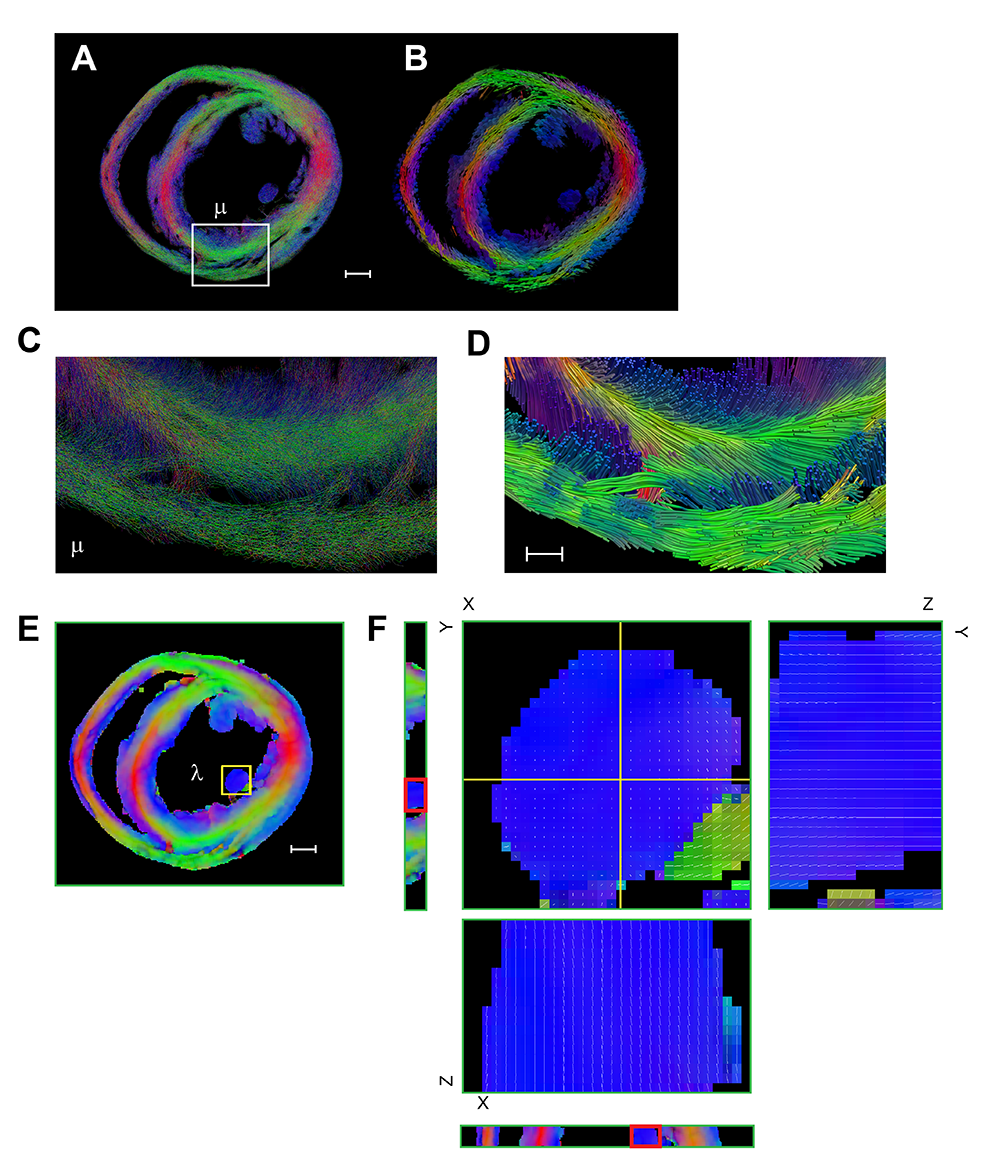


**Figure S12.** Skeletonized vascular perspective (axial view) of a representative apical short-axis heart slice (A) together with its associated tomographic vector field representation of the primary eigenvector (B). Scale bar, 500 μm. (C,D) Magnified view of the area μ in (A) and corresponding tomographic tractogram. Scale bar, 200 μm. Colors encode directional information. (E) DEC map of the principal eigenvector. Scale bar, 500 μm. (F) Magnified DEC map of the region labeled λ in (E) corresponding to the papillary muscles with overlayed hedgehogs, representing the length of the primary eigenvector projection along the vertical axis.


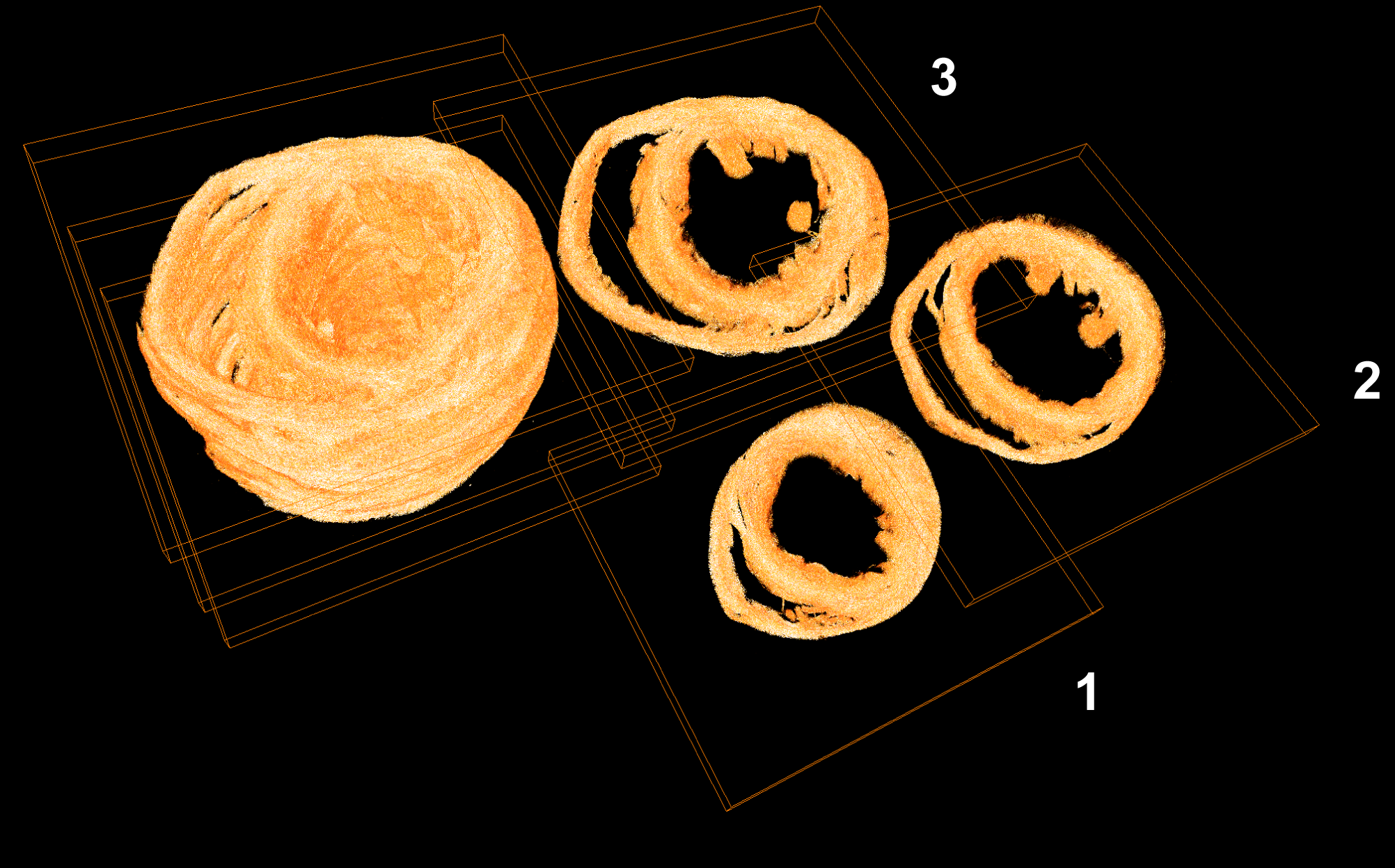


**Figure S13.** Axial planes within the heart where the axial slices shown in Fig. 4, S9, S10, S11, and S12 have been taken. Plane 1: Fig. 4a-l, Fig. S9a, Fig. S10a, Fig. S11. Plane 2: Fig. 4m,n,p, Fig. S9b, Fig. S10h. Plane 3: Fig. S10n, Fig. S12.


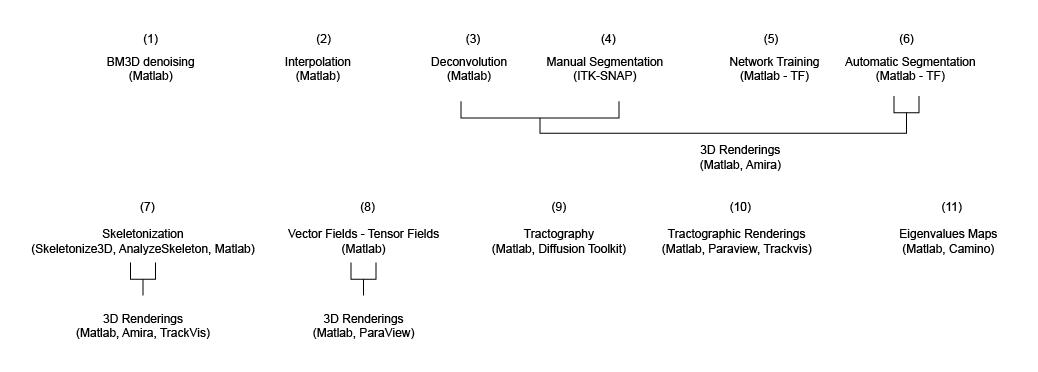


**Figure S14.** Flowchart describing the elaboration and visualization process complete with a description of the used visualization tools.

SUPPLEMENTARY REFERENCES

**73.** Tainaka, K. et al. Whole-body imaging with single-cell resolution by tissue decolorization. Cell 159, 911-924 (2014).

**74.** Cook, P. et al. in 14th scientific meeting of the international society for magnetic resonance in medicine. 2759 (Seattle WA, USA).

**75**. Wang, R., Benner, T., Sorensen, A. G. & Wedeen, V. J. in Proc Intl Soc Mag Reson Med. (Berlin).

**76.** Henderson, A. Paraview guide, a parallel visualization application. kitware inc.(2005). URL http://www. paraview. org.

**77.**  Yushkevich, P. A. et al. User-guided 3D active contour segmentation of anatomical structures: significantly improved efficiency and reliability. Neuroimage 31, 1116-1128 (2006).

**78.** www.python.org.

**79.** Abadi, M. et al. in OSDI. 265-283.

**80.** Dabov, K., Foi, A., Katkovnik, V. & Egiazarian, K. Image denoising by sparse 3-D transform-domain collaborative filtering. IEEE T. Image Process. 16, 2080-2095 (2007).

**81.** Bates, R. et al. Segmentation of vasculature from fluorescently labeled endothelial cells in multi-photon microscopy images. IEEE Trans. Med. Imaging 38, 1-10 (2017).

**82.** Dice, L. R. Measures of the amount of ecologic association between species. Ecology 26, 297-302 (1945).

**83.** Taha, A. A. & Hanbury, A. Metrics for evaluating 3D medical image segmentation: analysis, selection, and tool. BMC Med. Imaging 15, 29 (2015).

**84.** Milletari, F., Navab, N. & Ahmadi, S.-A. in 3D Vision (3DV), 2016 Fourth International Conference on. 565-571 (IEEE).

**85.** Lee, T.-C., Kashyap, R. L. & Chu, C.-N. Building skeleton models via 3-D medial surface axis thinning algorithms. CVGIP: Graphical Models and Image Processing 56, 462-478 (1994).

**86.** Arganda‐Carreras, I., Fernández‐González, R., Muñoz‐Barrutia, A. & Ortiz‐De‐Solorzano, C. 3D reconstruction of histological sections: application to mammary gland tissue. Microsc. Res. Techniq. 73, 1019-1029 (2010).

**87.** Mekkaoui, C. et al. Myocardial Scar Delineation Using Diffusion Tensor Magnetic Resonance Tractography. Journal of the American Heart Association 7, e007834 (2018).

**88.** Nielles-Vallespin, S. et al. Assessment of myocardial microstructural dynamics by in vivo diffusion tensor cardiac magnetic resonance. Journal of the American College of Cardiology 69, 661-676 (2017).

**89.** Streeter, D. D., Spotnitz, H. M., Patel, D. P., Ross, J. & Sonnenblick, E. H. Fiber orientation in the canine left ventricle during diastole and systole. Circ. Res. 24, 339-347 (1969).
